# Molecular Epidemiology and Clinical Impact of *Acinetobacter calcoaceticus-baumannii* Complex in a Belgian Burn Wound Center

**DOI:** 10.1371/journal.pone.0156237

**Published:** 2016-05-25

**Authors:** Daniel De Vos, Jean-Paul Pirnay, Florence Bilocq, Serge Jennes, Gilbert Verbeken, Thomas Rose, Elkana Keersebilck, Petra Bosmans, Thierry Pieters, Mony Hing, Walter Heuninckx, Frank De Pauw, Patrick Soentjens, Maia Merabishvili, Pieter Deschaght, Mario Vaneechoutte, Pierre Bogaerts, Youri Glupczynski, Bruno Pot, Tanny J. van der Reijden, Lenie Dijkshoorn

**Affiliations:** 1 Laboratory for Molecular and Cellular Technology, Queen Astrid Military Hospital, Brussels, Belgium; 2 Burn Wound Center, Queen Astrid Military Hospital, Brussels, Belgium; 3 Hospital Hygiene and Infection Control Team, Queen Astrid Military Hospital, Brussels, Belgium; 4 Clinical Laboratory, Queen Astrid Military Hospital, Brussels, Belgium; 5 Medical Communication and Information Systems, ACOS WB/Health Division, Queen Astrid Military Hospital, Brussels, Belgium; 6 Laboratory Bacteriology Research, University of Ghent, Ghent, Belgium; 7 Laboratoire de Bactériologie, CHU Mont-Godinne, Université Catholique de Louvain, Yvoir, Belgium; 8 Applied Maths, Sint-Martens-Latem, Belgium; 9 Department of Infectious Diseases C5-P, Leiden University Medical Center, Leiden, The Netherlands; Tianjin University, CHINA

## Abstract

Multidrug resistant *Acinetobacter baumannii* and its closely related species *A*. *pittii* and *A*. *nosocomialis*, all members of the *Acinetobacter calcoaceticus-baumannii* (Acb) complex, are a major cause of hospital acquired infection. In the burn wound center of the Queen Astrid military hospital in Brussels, 48 patients were colonized or infected with Acb complex over a 52-month period. We report the molecular epidemiology of these organisms, their clinical impact and infection control measures taken. A representative set of 157 Acb complex isolates was analyzed using repetitive sequence-based PCR (rep-PCR) (DiversiLab) and a multiplex PCR targeting OXA-51-like and OXA-23-like genes. We identified 31 rep-PCR genotypes (strains). Representatives of each rep-type were identified to species by *rpoB* sequence analysis: 13 types to *A*. *baumannii*, 10 to *A*. *pittii*, and 3 to *A*. *nosocomialis*. It was assumed that isolates that belonged to the same rep-type also belonged to the same species. Thus, 83.4% of all isolates were identified to *A*. *baumannii*, 9.6% to *A*. *pittii* and 4.5% to *A*. *nosocomialis*. We observed 12 extensively drug resistant Acb strains (10 *A*. *baumannii* and 2 *A*. *nosocomialis*), all carbapenem-non-susceptible/colistin-susceptible and imported into the burn wound center through patients injured in North Africa. The two most prevalent rep-types 12 and 13 harbored an OXA-23-like gene. Multilocus sequence typing allocated them to clonal complex 1 corresponding to EU (international) clone I. Both strains caused consecutive outbreaks, interspersed with periods of apparent eradication. Patients infected with carbapenem resistant *A*. *baumannii* were successfully treated with colistin/rifampicin. Extensive infection control measures were required to eradicate the organisms. *Acinetobacter* infection and colonization was not associated with increased attributable mortality.

## Introduction

The genus *Acinetobacter* currently comprises 49 validly named species (April 2016) including 5 novel proteolytic and haemolytic species (http://www.bacterio.net/acinetobacter.html) [1]. Some species are closely related, including the members of the so-called *Acinetobacter calcoaceticus-Acinetobacter baumannii* (Acb) complex [2, 3] and the 5 recently described species [1]. Apart from the 49 species, several *Acinetobacter* strains or groups of strains have been described with presumptive names, some of which are likely to represent potential novel species including ‘between 1 and 3’ [4] and ‘NB14’ [5]. Bacteria of the genus *Acinetobacter* are ubiquitous and are found in soil and water, insects, and in specimens from human and animal origin [6]. The closely related species *A*. *baumannii*, *A*. *pittii*, and *A*. *nosocomialis* of the Acb complex are most frequently isolated from human specimens and are important nosocomial pathogens [2, 7]. Identification of *Acinetobacter* species according to the current taxonomy and using commercial identification systems in the clinical microbiology laboratory is difficult. Meanwhile, *A*. *baumannii* has emerged as a major multidrug-resistant (MDR) microorganism in hospitals worldwide. It is one of the six ESKAPE organisms, which cause the majority of hospital-acquired infections and are able to *escape* current antibiotics [8]. *A*. *baumannii* is rarely recovered from the natural environment and infection reservoirs outside human and veterinary hospitals have not been identified [9]. Molecular typing of isolates obtained from hospitals and cities in Europe permitted the identification of 3 clonal lineages of *A*. *baumannii* that were associated with numerous outbreaks and were named EU clones I, II and III [10, 11]. By multilocus sequence typing (MLST), these clones were allocated to the corresponding clonal complexes 1–3 [12]. These clones have been found worldwide and, are also referred to as worldwide (WW) clones 1–3, while other widespread clones and MLST types have also been identified (http://pubmlst.org/abaumannii/) [13–15]. *A*. *baumannii* is responsible for a variety of infections, including wound infections, ventilator-associated pneumonia and bloodstream infections. Some studies provided evidence that *A*. *baumannii* infections are associated with increased mortality [16]. Carbapenems have long been the antimicrobial agents of choice, but today carbapenem resistance occurs frequently in *A*. *baumannii*, and even resistance to colistin, the last resort antibiotic, has been reported [17]. Their capacity for long-term survival in the hospital combined with antibiotic resistance and a potential to dynamic genomic reorganization under selective pressure [18], make *A*. *baumannii* and, to a lesser extent, the closely related species *A*. *pittii* and *A*. *nosocomialis* [19] important nosocomial pathogens. Patients vulnerable to MDR *A*. *baumannii* infection are critically ill patients as found in intensive care units. Special categories are patients with trauma e.g., due to accidents, natural disasters or military actions, or burn patients. Outbreaks of *A*. *baumannii* isolations were observed among service members injured in the Iraq/Kuwait (Operation Iraqi Freedom) and in Afghanistan (Operation Enduring Freedom) [20–26]. Although modern medical care has significantly reduced the mortality among thermally injured, the burn wound is still a site of increased susceptibility to opportunistic colonization and subsequent infection remains a major issue of concern [27]. MDR *A*. *baumannii* is increasingly associated with these infections [28, 29].

In November 2006, a severely burned woman was transferred from Algiers (Algeria) to the burn wound center (BWC) of the Queen Astrid military hospital in Brussels (Belgium). Bacteriological analysis showed that the patient’s wounds and nasopharynx were colonized with extensively drug-resistant (XDR) Acb complex (resistant to carbapenems), which spread to other patients. Despite drastic infection control measures, this first outbreak was followed by recurrent episodes of epidemic spread. Here we report the in-depth epidemiological investigation of Acb complex colonization in the BWC, prompted by this first carbapenem-resistant *A*. *baumannii* (CRAB) outbreak. During a 52-month period, 48 patients were colonized or infected with members of the Acb complex. Isolates were presumptively identified and investigated for antibiotic susceptibility using the VITEK 2 microbial identification (ID) and antibiotic susceptibility testing (AST) systems. Representative isolates were further characterized at strain and species level using a combination of DNA-based methods. In addition, the strains were investigated for the occurrence of OXA-51-like and OXA-23-like oxacillinase and other genes. Results were interpreted in light of the time-space origin of isolates for analysis of the epidemiology of Acb complex strains in the BWC. Furthermore, the clinical impact of the organisms and infection control measures were evaluated. This study adds novel information with regard to the occurrence of both baumannii and non-baumannii *Acinetobacter* species in a burn wound setting.

## Materials and Methods

### Setting

The Brussels BWC is a department of the Queen Astrid military hospital and functions as a referral center for Belgium. It was built in 1980 and consists of an intensive care unit (ICU), a medium care unit (MCU), and consultation and ambulatory services. The ICU harbored one admission room, eight single-bed rooms with an average occupancy rate of 70% and three bathrooms. The medium care unit harbored 12 double-bed rooms and two bathrooms. All bathrooms were equipped with a balneotherapy facility and 2 ICU rooms and 1 bathroom were equipped with a laminar airflow installation. Hand washing with a detergent skin cleansing solution (Hibiscrub, Zeneca Pharma, Cergy, France) was required between patient contacts. Masks, caps, and gloves were required at all times in the patient rooms and bathrooms. Sterile gloves were required for wound care procedures. Microbiological screening samples were taken at admission, every second day (ICU), twice a week (MCU) or whenever deemed necessary for clinical reasons. Clinical samples consisted of swabs of the wound areas, the nasopharynx and the peri-anal region, urine, expectoration, blood and catheter samples, if applicable. Annually, 300–400 patients were admitted to the BWC, while more than 10,000 consultations were performed each year. Available data, derived from existing medical or administrative files, from all patients admitted to the ICU and MCU of the BWC between November 2006 and February 2011 was analyzed in this retrospective epidemiological investigation, which was coordinated by the Hospital Infection Control Team and the Laboratory for Molecular and Cellular Technology, the BWC supporting research unit. Since data collection required no contact with the patients and no clinical samples other than those relevant for the treatment were collected from patients (only from the environment), this non-interventional retrospective data analysis is not subjected to the Belgian law concerning experiments on the human person (May 7, 2004) and ethics committee approval is not required. Health care professionals consulted and processed the data for their own use, respecting the provisions on professional secrecy. Patient records/information was anonymized and de-identified prior to analysis. Under these conditions, no informed consent of the patients is required.

### Definitions

For patients from whom Acb complex strains were recovered on bacterial culture, medical records were further screened for laboratory and clinical signs of infection. The French Society for Burn Injuries (SFETB) diagnostic criteria for infection in burn patients (http://www.sfetb.org/index.php?rub=textes-officiels&art=doc_ref_8) were used for diagnosis of Acb complex pneumonias, bloodstream infections, burn wound infections and urinary tract infections. In the absence of clear laboratory and clinical signs of infection, patients from whom a positive bacterial culture was obtained, were considered colonized. Colonization on admission was defined as the isolation of an organism within 24 h of their hospitalization. Cross-colonization was defined as the isolation of genotypically identical isolates from patients who were in the same ward of the BWC at the same time. Isolates that were non-susceptible to at least one agent in three or more antimicrobial categories were considered MDR, while extensively or extremely drug resistant (XDR) was defined as non-susceptibility to at least one agent in all but two or fewer antimicrobial categories [30].

### Infection control and interventions

Several interventions were made in response to the sudden increased incidence of *Acinetobacter* colonization in the BWC. In the first place, patients were systematically screened for *Acinetobacter* on admission (wounds, nasopharynx, groin and peri-anal region), colonized patients were isolated in private rooms with dedicated personnel and equipment, a dedicated infection control task force was set up, an epidemiological investigation was initiated and infection control seminars (with an emphasis on hand hygiene, using alcohol-based hand rubs) were organized. Rapidly, the infection control task force started to realize that these initial measures were insufficient to contain *Acinetobacter* and on January 11, 2007, the BWC was closed (the closure was announced in a press release) for deep cleaning and specialized decontamination using vaporized hydrogen peroxide (STERIS Corporation, Brussels, Belgium). Admissions were stopped and resident patients were transferred to a back up ICU (initially reserved for disaster response). The BWC was reopened on February 15, 2007, after surface and air samples were found to be negative for Acb complex. In December 2009, a new burn unit, designed and constructed to allow for extensive infection control measures, was put into operation. Each of the 8 new ICU rooms was equipped with a laminar airflow, dedicated personnel and material entry and exit airlocks, an integrated balneotherapy facility and dedicated medical equipment.

### The first outbreak

On November 21, 2006, a 29-year old woman was transferred from the military hospital in Algiers to the BWC of the Queen Astrid military hospital in Brussels. Eight days earlier, she had been severely burned (33% TBSA burned, most 3^rd^ degree) after a gas explosion in her kitchen. Routine bacteriological analysis, performed immediately upon arrival at the BWC in Brussels, showed that the patient’s burn wounds and nasopharynx were colonized with XDR Acb complex, resistant to carbapenems and susceptible to only colistin. When two additional burn wound patients were found to be colonized with XDR *Acinetobacter*, a limited epidemiological investigation was initiated. This investigation, which was mainly based on repetitive sequence-based PCR fingerprinting (rep-PCR, DiversiLab) of routine Acb complex isolates, revealed that the 3 patients were colonized with an identical CRAB strain. Previously, *Acinetobacter* was sporadic in the BWC and no carbapenem resistance had ever been observed. This first CRAB outbreak prompted a clinically indicated and partially retrospective epidemiological investigation of Acb complex colonization and infection in the BWC.

### Epidemiological investigation

Medical and microbiological records of patients that were colonized/infected with Acb complex were reviewed. The characteristics of the colonized patients were compared with those of the general BWC population. No clinical samples other than those that were relevant for the treatment were taken from patients. Environmental sampling was performed on January 12 and 22, February 9, April 3, 11 and 20, May 10, 11, 14 and 23 and June 18, 2007. In total, 61 (55%) of the 111 health care workers of the BWC, including 29 nurses, 8 physiotherapists, 7 cleaning staff members and 7 MDs, were screened (nose, throat, forehead and peri-anal swabs) on a voluntary basis for transient carriage of *Acinetobacter*. Environmental samples consisted of 120 swabs of multiple surfaces within the patient environment (including floors, bed sheets, bed side tables, trolleys, sinks, respirators, air conditioning aspiration grids, hemofiltration machines and computer keyboards), 13 air samples (100L) aspirated using a MAS-100 microbial air monitoring system (MBV AG, Stäfa, Switzerland) and tap water samples from all faucets in the BWC (faucet spouts were disinfected with a flame and water was allowed to run for two to three minutes before sampling in sterile 1L bottles). The water feeding the hydrotherapy facilities was sampled every 2 months (routine).

### Routine microbiological analysis

Bacteria were isolated from clinical and environmental samples using standard microbiological procedures as described in the Manual of Clinical Microbiology (editions 8–10, issued by the American Society for Microbiology). In short, blood agar, McConkey and mannitol salt agar plates (all purchased from bioMérieux, Brussels, Belgium) were inoculated and incubated overnight at 37°C. Colonies were identified using the gram-negative (GN) and gram-positive (GP) identification cards for the VITEK 2 microbial ID system (bioMérieux, Brussels, Belgium), according to the manufacturer’s instructions.

### Reference strains

Five Acb complex reference strains were received from Leiden University Medical Center (LUMC), the Netherlands: LUH 09695 (*A*. *pittii*), LUH 05988 (*A*. *nosocomialis*), RUH 0875 (EU clone I), RUH 0134 (EU clone II), and LUH 05875 (EU clone III). A representative of the *A*. *baumannii* ‘T strain’ (NCTC 13423) was obtained from the Central Public Health Laboratory, London, UK [31].

### Antibiotic susceptibility testing

The VITEK 2 AST system (bioMérieux, Brussels, Belgium) was used to determine antibiotic susceptibilities (using AST-N155 or AST-N158 cards) of the Acb complex isolates (as identified by the VITEK 2 microbial ID system) and reference strains, according to the manufacturer’s recommendations. The Advanced Expert System (AES) (software versions 02.01 to 05.03) was used to analyze the raw antibiotic susceptibility data. Clinical interpretation of minimum inhibitory concentration (MIC) values of antibiotics was based on the Clinical and Laboratory Standards Institute (CLSI) breakpoints.

### Typing and species identification by molecular methods

The organisms were characterized at different levels by three methods, i.e. at strain level by rep-PCR, at MLST level by sequencing of seven house keeping gene fragments, and at species level by *rpoB* sequencing. Of these methods, rep-PCR has the highest resolution and can, in local situations, be used to distinguish strains. MLST has less discriminatory power, but the sequence types can be compared to Internet based databases to assess the global or regional spread of particular MLST types/clones or clonal complexes. Sequence analysis of zones 1 and 2 of the *rpoB* gene has been proven to be a reliable method for species identification [2].

For rep-PCR, genomic DNA was extracted from bacterial colonies using the UltraClean^TM^ Microbial DNA Isolation Kit (MoBio Laboratories, Carlsbad, CA). PCR amplification was performed using the DiversiLab Acinetobacter 3.4 fingerprinting kit (bioMérieux, Marcy L’Etoile, France), according to the manufacturer’s instructions. Rep-PCR products were separated by electrophoresis on microfluidic chips and analyzed with the Agilent 2100 Bioanalyzer. XML files of pattern sets produced by the web-based DiversiLab software (bioMérieux) were imported into BioNumerics biological data analysis software (version 6.5, Applied Maths, Sint-Martens-Latem, Belgium) for autocorrection (fingerprint edges not fixed; maximum shift: 5.0%; maximum stretch compression: 3.0%; number of iterations: 5), similarity (Pearson correlation coefficient; optimization: 5%; curve smoothing: 0%; negative similarities: clip to zero) and cluster analysis (UPGMA; active zones: 0.0% - 100.0%). As validated previously [32, 33], isolates with band patterns with ≥95% similarity were considered as belonging to the same strain.

MLST analysis was performed as previously described [12]. This approach is based on the sequencing of internal regions of seven housekeeping genes. The *Acinetobacter* MLST database was at the time of analysis located and maintained at the Institut Pasteur (http://www.pasteur.fr/mlst), but has since been moved to http://bigsdb.readthedocs.org/en/latest/submissions.html.

For *rpoB* gene analysis, the partial sequence of the *rpoB* gene, i.e. zone 1+2 (861 bp) [34] was determined as previously described [2]. The obtained gene sequences were analysed using BioNumerics software (version 5.1) (Applied Maths, Sint-Martens-Latem, Belgium) and compared to a library of sequences of the validly described *Acinetobacter* species obtained from EMBL and from reference strains of the LUMC collection.

### PCR detection of antibiotic resistance determinants

OXA-51-like and OXA-23-like oxacillinase genes were identified by multiplex PCR as previously described [35, 36]. Epidemic strains (i.e. strains assumed to be involved in cross infection/epidemic spread) were further screened for the presence of acquired OXA carbapenemases (23, 24 and 58-like), extended spectrum β-lactamases (ESBLs) (AmpC, BEL, GES, VEB and PER) and metallo-β-lactamases (VIM, IMP and NDM) as previously described [37]. The ISAba1 to 4 insertion sequences and the integron sequence were identified as previously described [38].

### 16S rRNA gene analysis

The 16S rRNA gene sequence was determined and analyzed as previously described [39]. Briefly, the complete 16S rRNA gene was amplified, followed by sequencing reactions using the Big Dye Terminator Sequencing kit (Applied Biosystems, Foster City, Calif.) and analysis of the obtained fragments on the ABI 310 capillary electrophoresis apparatus (Applied Biosystems). Total gene assembling of the obtained fragments, alignment, and clustering were done with GeneBase (Applied Maths, Sint-Martens-Latem, Belgium). The obtained sequences were compared to all known sequences in the GenBank by Blast (National Center for Biotechnology Information, Bethesda, Md.; http://www.ncbi.nlm.nih.gov/blast/index.html).

### Treatment of CRAB infections

CRAB infected patients received systemic colistin treatment, which consisted of 3 to 9 million units of colistimethate sodium per day for 5 days (urinary tract infections), 7 days (wound infections) or 10 days (respiratory tract infections). Additionally, infected or heavily colonized burn wounds were treated on a daily basis with a compounded colistin lotion prepared by the local hospital pharmacy, consisting of 0.5 g colistimethate sodium, 0.1 ml lactic acid, 5.7 g paraffin, 3.8 g Tween 80, 6.2 g cetylalcohol, 3.3 g Labrafil and 80 g of sterile water per 100 g. Because it was suggested that colistin’s activity on *A*. *baumannii* is increased in the presence of rifampin [40], a daily dose of 600 mg rifampicin was usually added.

## Results

### Patient characteristics

During the 52-month study period (November 2006 –February 2011), 1438 patients [mean age: 36.0 years (range: 0 to 97 years), mean total body surface area (TBSA) burned: 9.9% (range: 1 to 93%), mean duration of hospitalization: 9.2 days (range: 1 to 121 days)] were treated in the BWC. An overview of patients from whom Acb complex bacteria were cultured is given in Table 1. The bacteria were isolated from 48 patients (3.3%) [mean age: 46.0 years (range: 1 to 87 years), mean TBSA burned: 21.0% (range: 1 to 74%), mean duration of hospitalization: 39.0 days (range: 1 to 121 days)]. Seven patients (14.6%) were hospitalized in the BWC for wounds other than burn wounds (e.g. a spider bite). Acb complex colonization was detected after an average of 14.7 days (range: 0 to 48 days). The length of colonization was on average 13.3 days (range: 1 to 80 days). Eleven patients (22.9%) were colonized on admission; nosocomially acquired colonization was suspected in the remaining 37 patients (77.1%). Only 11 *Acinetobacter* infections (all *A*. *baumannii*) could clearly be diagnosed (positive culture + laboratory and clinical signs of infection) in 8 patients (16.7%): 7 burn wound infections, 3 pneumonias and 1 bloodstream infection (Table 1). Co-morbidity, mainly psychiatric disorders (*n* = 10) or diabetes (*n* = 6), was observed in 26 patients. All colonized patients were co-colonized or co-infected with other bacteria, mainly with *P*. *aeruginosa* (*n* = 22, 45.8%), methicillin-sensitive *Staphylococcus aureus* (MSSA; *n* = 15, 31.2%), methicillin-resistant *S*. *epidermidis* (MRSE; *n* = 13, 27.1%), methicillin-resistant *S*. *aureus* (MRSA; *n* = 11, 22.9%) or ESBL producing *Enterobacteriaceae* (*n* = 8, 16.7%) (Table 1).

**Table 1:** Demographics and clinical characteristics of patients with Acb complex (as identified by the VITEK 2 system) colonization or infection.

Patient	Age (yr)	Sex^a^	TBSA burned (%)^b^	Burn degree	Date of hospitalization (day/mo/yr)	Length of stay (days)	Length of stay before ABC isolation (days)	Suspected import from	Length of colonization/ infection (days)	Sequence of culture sites (No. of analyzed isolates)^c^	Infection^d^	Rep-PCR profiles^e^ (No. of isolates)	Co-colonizing/infecting microorganism^f^	Comorbidity	Outcome	Specific treatment
1	29	F	33	3	21/11/2006	46	0	Algeria	45	W(4), N(2), C(1), E(1)	BWI, pneumonia	**9**(4), **12**(2), **13**(1), **16a**(1)	*K*. *pneumoniae* (ESBL+), MRSA, *P*. *stuartii*, P. aeruginosa*		Died of *P*. *aeruginosa* sepsis	Colistin
2	22	F	Reconstruction	NA	27/11/2006	27	20		1	W(1)		*22(1)*	*S*. *aureus*, MRSE, *P*. *mirabilis*		Discharged	No
3	48	F	Reconstruction	NA	20/12/2006	37	31		1	W(1)		*22(1)*	*S*. *aureus*, MRSE*, *P*. *mirabilis*, *P*. *aeruginosa*		Discharged	Colistin/rifampicin
4	68	F	31	3	22/12/2006	16	3		11	E(1)	BWI	**13**(1)	P. aeruginosa*	Diabetes, ethylism, previous breastcancer, anxiety	Died of *P*. *aeruginosa* sepsis	Colistin/rifampicin
5	60	M	14	2	22/12/2006	53	10		43	T(1), W(1), N(1), E(2), U(1)		**13**(6)	*P*. *aeruginosa*	Diabetes, toxic inhalation, psychosis, renal insufficiency	Discharged	Colistin/rifampicin
6	87	F	15	3	28/12/2006	52	9		38	W(3), N(3), T(4), E(1)		**13**(10), **16b**(1)	P. aeruginosa*		Died of *P*. *aeruginosa* sepsis	Colistin/rifampicin
7	44	M	55	3	18/03/2007	20	13		3	W(4), T(1)	BWI	**13**(5)	MRSA	Toxic inhalation, suicidal	Died of MRSA sepsis	Colistin/rifampicin
8	83	F	65	2	23/04/2007	17	7		3	C(2), U(1), W(3), N(1)	BWI	**13**(7)	*E*. *coli*, *E*. *gallinarum*, *S*. *hominis*	Diabetes, hypertension	Discharged	Colistin/rifampicin
9	47	M	55	3	30/03/2007	76	37		37	W(7), N(4), T(1), E(3)	BWI, pneumonia	**12**(1), **13**(14)	*E*. *coli*, *P*. *mirabilis*, *E*. *cloacae*, *S*. *aureus*, *S*. *haemolyticus*, MRSE, *E*. *faecalis*, *C*. *albicans*	Peripheral vascular disease	Discharged	Colistin
10	70	F	74	2 and 3	22/04/2007	18	17		1	W(1)		**13**(1)	*S*. *aureus*, *P*. *mirabilis*, *E*. *coli*, *E*. *faecalis*, *S*. *marcescens*	Diabetes, psoriasis	Discharged	Colistin/rifampicin
11	2	M	8	2	11/06/2007	3	0	Belgium	1	W(1)		26(1)			Discharged	No
12	60	M	Necrotic fasciitis	NA	16/05/2007	121	41		80	W(9)	BWI	**12**(1), **13**(8)	*E*. *coli* (ESBL+), *S*. *aureus*, *P*. *aeruginosa*	Diabetes	Discharged	No
13	83	F	35	3	14/11/2007	91	8		60	W(6)		1(6)	MRSA, MRSE, *S*. *hominis* (OxaR), *E*. *cloacae*, *S*. *marcescens*, *P*. *aeruginosa*, *E*. *faecalis*, *C*. *tropicalis*	Suicidal	Died of a heart condition	Colistin/rifampicin
14	47	M	30	3	22/10/2007	45	35		10	W(1), T(2)		**13**(3)	MRSA, MRSE, *S*. *haemolyticus* (OxaR), *S*. *lugdunensis*, *E*. *cloacae*, *E*. *faecalis*, *C*. *tropicalis*	Depression	Discharged	Colistin/rifampicin
15	41	F	33	3	18/10/2007	93	48		32	W(5), T(1), V(1)		**13**(7)	MRSA, MRSE, *S*. *haemolyticus* (OxaR), *S*. *hominis* (OxaR), E. coli, E. cloacae, E. faecalis*, C. albicans	Astma, depression, smoker	Discharged	Colistin/rifampicin
16	76	M	2	2	5/02/2008	22	20		1	E(1)		1(1)	*S*. *aureus*	Inhalation injury	Discharged	No
17	80	F	20	3	19/01/2008	65	42		4	W(2)		**13**(2)	MRSE*, *E*. *coli*, *P*. *aeruginosa*, *S*. *warneri* (OxaR)	Kyphoscoliosis, rheumatoid arthritis	Died of culture negative sepsis	No
18	66	F	10	2	24/01/2008	120	48		64	E(1), W(3), N(1), F(2)	Pneumonia	**13**(7)	MRSA, MRSE, *P*. *stuartii*, *P*. *mirabilis*, *E*. *faecalis*, *P*. *aeruginosa*		Discharged	Colistin/rifampicin
19	70	F	10	3	14/03/2008	39	16		10	W(1), U(1)		**13**(2)	*S*. *aureus*, MRSE, *S*. *epidermidis*, *E*. *coli*, *E*. *faecalis*, *K*. *pneumoniae*, *P*. *aeruginosa*	Cardio vascular accident	Discharged	No
20	1	M	2	2	23/06/2008	12	17		1	W(1)		*23(1)*	*S*. *aureus*, *E*. *faecalis*, *K*. *oxytoca*		Discharged	No
21	2	M	8	2	1/09/2008	2	1		1	W(1)		*23(1)*	*S*. *saprophyticus*		Discharged	No
22	2	F	15	2	14/11/2008	17	14		1	W(1)		*10(1)*	*S*. *aureus*, *S*. *epidermidis*, *E*. *coli*, *Enterobacter spp*., *S*. *maltophilia*		Discharged	No
23	51	F	Lyell syndrome	NA	2/01/2009	12	4	Tunisia	3	T(2), W(3), V(1)		**3**(1), **12**(5)	MRSA, MDR *P*. *aeruginosa*, *C*. *albicans*, *C*. *glabrata*		Died of *P*. *aeruginosa* pneumonia	No
24	43	F	5	2 and 3	30/01/2009	16	0		1	W(1)		**3**(1)	MRSA	Depression, ethylism, smoker	Discharged	No
25	69	M	4	2	18/01/2009	24	16		8	W(1)		*31(1)*	MRSA	Diabetes, pyrosis, renal insufficiency	Discharged	No
26	73	M	5	2	5/02/2009	93	14		34	W(4)		**12**(4)	MRSA, *P*. *aeruginosa*		Discharged	No
27	29	M	52	2	27/02/2009	39	1		10	W(3), B(1)	BSI, BWI	**12**(4)	*Enterobacter spp*., *P*. *aeruginosa*	HIV infection	Discharged	No
28	56	F	31	2	22/02/2009	44	20		4	W(1)		**12**(1)	K. pneumoniae, S. marcescens*, C. albicans, C. glabrata, P. aeruginosa	Obesity	Died of *S*. *marcescens* sepsis	No
29	35	M	12 (plant contact)	2	11/05/2009	38	0		1	W(1)		*31(1)*	*K*. *pneumoniae*, *S*. *haemolyticus*, *S*. *lugdunensis*, *S*. *warneri*, *E*. *coli*, *E*. *cloacae*, *P*. *mendocina*, *P*. *mirabilis*		Discharged	No
30	48	M	13	2	18/06/2009	6	0	Belgium	1	W(1)		2(1)	*P*. *aeruginosa*, *K*. *pneumoniae*		Discharged	No
31	60	M	8	2 and 3	25/06/2009	16	8		1	W(1)		*28(1)*	*P*. *aeruginosa*, MRSE, *A*. *lwoffii*	Depression, heart surgery	Discharged	No
32	30	M	9	2	22/07/2009	15	0		1	T(1)		*29(1)*	*Raoultella planticola*, *S*. *aureus*, *Pantoea agglomerans*, *S*. *haemolyticus*		Discharged	No
33	1	M	13	2	7/09/2009	28	15		1	T(1)		*20(1)*	*E*. *coli* (ESBL+), *P*. *aeruginosa*, *S*. *aureus*, *P*. *mirabilis*, *E*. *faecalis*		Discharged	No
34	3	F	1	3	18/09/2009	19	11		1	T(1)		*28(1)*	*E*. *cloacae* (ESBL+)		Discharged	No
35	13	M	Reconstruction	NA	7/12/2009	31	41		2	W(1)		*21(1)*	*S*. *aureus*, *S*. *epidermidis*		Discharged	No
36	49	M	Spider bite	NA	28/02/2010	70	13		1	W(1)		**3**(1)	MRSA, *S*. *epidermidis*, *B*. *cepacia*, *P*. *putida*		Discharged	No
37	56	M	27	2 and 3	7/05/2010	50	0	Tunisia	15	W(8)		**11**(3), **12**(2), **14**(1), **15**(2)	MRSE, *E*. *coli*, *P*. *aeruginosa*, *E*. *cloacae* (ESBL+), *Bacillus spp*.	Smoker	Discharged	No
38	54	M	1	2	21/05/2010	1	0	Belgium	1	W(1)		*33(1)*	*S*. *maltophilia*		Discharged	No
39	24	F	1	3	21/07/2010	6	5		1	W(1)		*34(1)*	MRSE, *E*. *coli*, *E*. *cloacae*, *P*. *aeruginosa*	Psychosis	Discharged	No
40	3	F	11	2	29/07/2010	2	0	Morocco	1	W(1)		**6**(1)	S. haemolyticus*		Discharged	No
41	1	M	8	2	22/08/2010	6	2		3	T(1)		26(1)	*E*. *coli* ESBL+, *E*. *cloacae*, *P*. *aeruginosa*		Discharged	No
42	64	F	7	2	19/08/2010	14	9		6	W(2)		*30(2)*	*S*. *aureus*, *E*. *coli*	Smoker, ethylism	Discharged	No
43	73	M	19	2 and 3	24/07/2010	65	46		1	W(1)		*25(1)*	*E*. *cloacae*, *Citrobacter spp*., *S*. *aureus*	Smoker	Discharged	No
44	49	M	50	2 and 3	30/09/2010	56	14		23	N(1), W(2), T(3)		***17(4)*, *18(2)***	*K*. *pneumoniae*, MRSE, *P*. *mirabilis*, *S*. *maltophilia*, *Trichosporon asahii*, *Aeromonas hydrophilia*, *E*. *aerogenes*, *M*. *morganii*, *C*. *krusei*, *C*. *glabrata*	Smoker, hernia	Discharged	No
45	53	M	42	3	3/11/2010	100	29		19	T(1)		*27(1)*	*E*. *coli*, *K*. *pneumoniae*, *P*. *mirabilis*, *S*. *maltophilia*	Depression, suicidal	Discharged	No
46	37	F	25	2 and 3	11/12/2010	35	0	Tunisia	20	W(2)		**7**(1), **12**(1)	S. marcescens*, E. coli (ESBL+), E. cloacae*, K. pneumoniae (ESBL+), *S*. *maltophilia*, *P*. *aeruginosa*		Discharged	No
47	70	M	2 (frost bite)	3	13/01/2011	33	0	Belgium	1	W(1)		*8(1)*	*S*. *aureus*, *S*. *marcescens*, *E*. *aerogenes*, *E*. *cloacae*, *C*. *tropicalis*, *P*. *vulgaris*	Smoker	Discharged	No
48	74	F	Gangrene	NA	10/02/2011	61	20		28	W(1)		*32(1)*	*C*. *albicans*, *C*. *krusei*, *K*. *pneumoniae*	Breast cancer	Discharged	No
	46,0	22F	21,0			39,0	14,7		13,3				* *		16.7% mortality	

^a^ F, female; M, male.

^b^ TBSA, total body surface area.

^c^ W, wound swab; N, nose swab; V, vaginal swab; C, catheter; T, throat swab; E, expectoration; B, blood; U, urine; F, faeces.

^d^ BSI, bloodstream infection; BWI, burn wound infection.

^e^ Rep-PCR profiles harboring carbapenem resistant isolates are indicated in bold. Non-baumannii *Acinetobacter* strains are indicated in italics.

^f^ ESBL+, extended spectrum beta-lactamase positive (as identified by the VITEK 2 system)

MRSA, methicillin-resistant *Staphylococcus aureus*; MRSE, methicillin-resistant *Staphylococcus epidermidis*; OxaR, oxacillin resistance; MDR, multidrug-resistant

*, isolated from haemoculture.

### Epidemiological investigation

**Fig 1:**
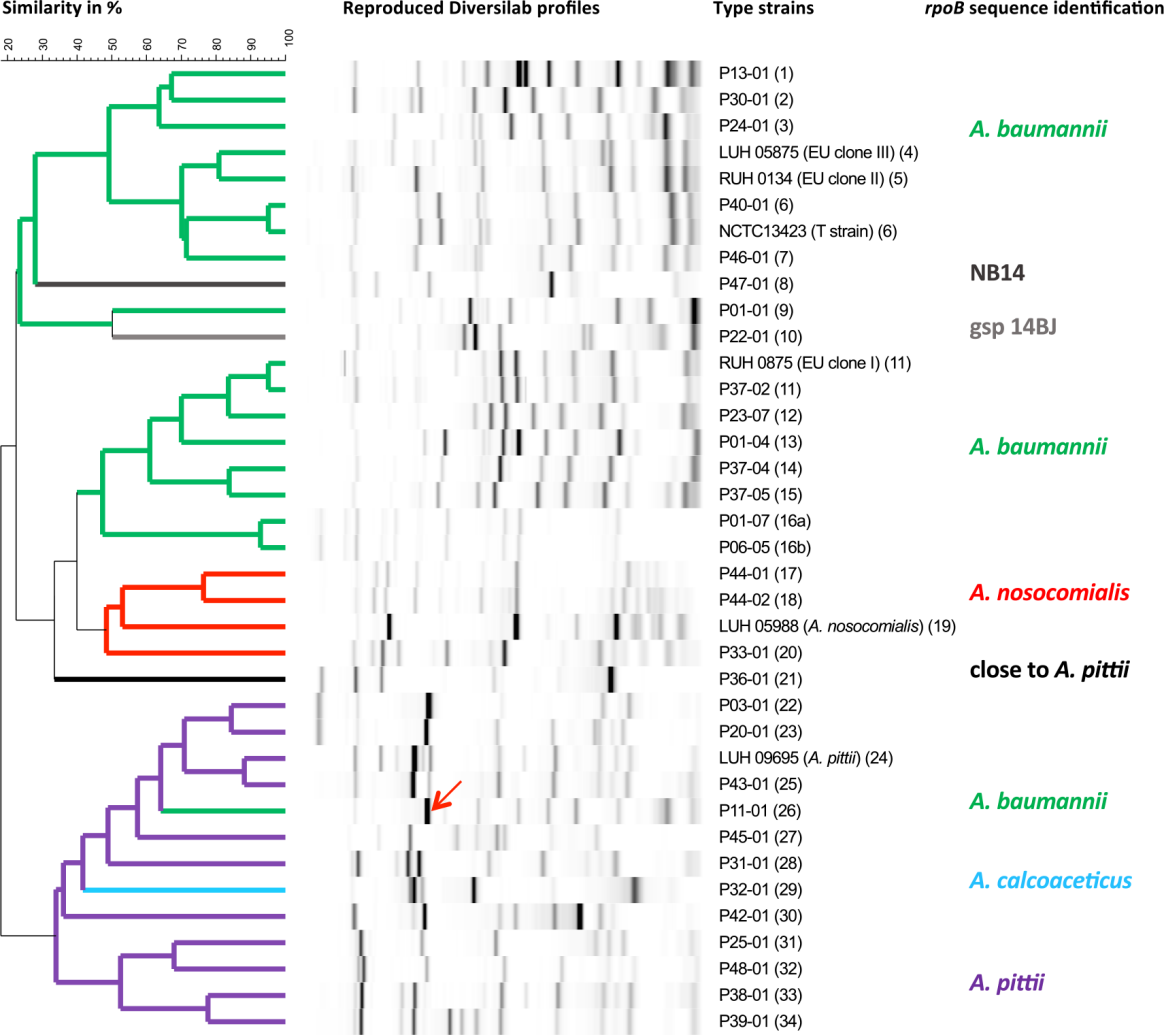
Dendrogram based on UPGMA and Pearson correlation coefficient, representing genetic similarity among reference and clinical Acb complex isolates. Representative Acb complex isolates (Type strains) were typed by rep-PCR (DiversiLab) and analyzed (autocorrection, Pearson correlation coefficient, optimization and curve smoothing) with the BioNumerics DiversiLab plugin. Thirty-one rep-PCR type strains with <95% similarity and 6 reference strains are shown. Different colors indicate distinct species within the *Acinetobacter* genus. The red arrow indicates the band responsible for the clustering of *A*. *baumannii* strain P11-01 in the *A*. *pittii* cluster (not uncommon for Pearson correlations).

No *Acinetobacter* spp. were isolated from healthcare workers, water or air samples, but 12 *A*. *baumanni* isolates were cultured from the patients’ environment (respirators, bed side tables, trolleys, mattresses, air conditioning aspiration grids and the lever and the keyboard of a haemodynamic monitor). A total of 157 Acb complex isolates, consisting of 149 clinical and 8 environmental isolates, which were kept in the bacterial culture collection of the clinical laboratory, were selected for epidemiological analysis using DNA-based approaches. The isolates included at least one isolate from each colonized site of each colonized patient and the hospital environment. The clinical isolates were from wound swabs (predominant site, *n* = 95), throat swabs (*n* = 20), nasal swabs (*n* = 13), sputum (*n* = 10), urine (*n* = 3), catheter swabs (*n* = 3), vaginal swabs (*n* = 2), feces (*n* = 2) and blood (*n* = 1). Of the 157 Acb complex isolates, 131 (83.4%) were identified to *A*. *baumannii*, 15 (9.6%) to *A*. *pittii*, 7 (4.5%) to *A*. *nosocomialis*, 1 (0.6%) to *A*. *calcoaceticus* and 3 (1.9%) to potentially novel species. The 26 non-baumannii *Acinetobacter* isolates were exclusively isolated from wounds (n = 18) and nose/throat (n = 8). None-baumannii *Acinetobacter* strains were never isolated from expectoration, urine, blood or catheters. Rep-PCR analysis of the 157 selected *Acinetobacter* isolates revealed, at the 95% cut-off level, 31 genotypes among the *Acinetobacter* isolates (Fig 1 and Table 2). Rep-PCR genotypes 4, 5, 19 and 24 were only observed in single reference strains. Rep-PCR profiles 16a and 16b were closely related (92% similarity) (Fig 1).

**Table 2:** Characteristics of 157 Acb complex isolates (as identified by the VITEK 2 system) from 48 patients admitted to the burn unit over a 52-month period and of 6 reference strains.

REP-PCR profile (Fig 1)	Type or reference strain (in bold)	No. of isolates	No. of patients	Identification based on *rpoB* sequence (Fig 2)	MLST for a selection of strains									*bla*_OXA-51-like_	*bla*_OXA-23-like_	Antibiotic susceptibility			
					ST (No. of isolates)	CC	Allele									β-lactam	Amino-glycoside	Quino-lone	Carba-penem
							*cpn60*	*fusA*	*gltA*	*pyrG*	*recA*	*rplB*	*rpoB*						
1	P13-01	7	2	*A*. *baumannii*	309(1)		12	1	2	2	9	1	5	positive	negative	R	S	R (2/7)	S
2	P30-01	1	1	*A*. *baumannii*	314(1)		25	1	18	1	56	2	14	positive	negative	R	S	S	S
3	P24-01	3	3	*A*. *baumannii*	311(1)		3	1	2	1	11	2	4	positive	positive	R	R	R	R
4	**LUH 05875 (EU clone III)**			*A*. *baumannii*	3(1)	CC3	3	3	2	2	3	1	3	positive	negative	R	R	R	R
5	**RUH 0134 (EU clone II)**			*A*. *baumannii*	2(1)	CC2	2	2	2	2	2	2	2	positive	negative	R	R	R	S
6	P40-01	1	1	*A*. *baumannii*	2(1)	CC2	2	2	2	2	2	2	2	positive	negative	R	R	R	S
6	**NCTC 13423 (T strain)**			*A*. *baumannii*	2(1)	CC2	2	2	2	2	2	2	2	positive	negative	R	R	R	R
7	P46-01	1	1	*A*. *baumannii*	2(1)	CC2	2	2	2	2	2	2	2	positive	negative	R	R	R	R
8	P47-01	1	1	NB14										negative	negative	R	S	S	S
9	P01-01	4	1	*A*. *baumannii*	10(1)	CC10	1	3	2	1	4	4	4	positive	positive	R	R	R (1/4)	R
10	P22-01	1	1	*A*. *courvalinii* sp. nov.										negative	negative	R	S	S	S
11	**RUH 0875 (EU clone I)**			*A*. *baumannii*	1	CC1	1	1	1	1	5	1	1	positive	negative	R	R	R	S
11	P37-02	3	1	*A*. *baumannii*	315(1)	CC1	1	56	1	1	5	1	1	positive	positive	R	R	R	R
12	P23-07	21	9	*A*. *baumannii*	1(1) or 310(1)	CC1	1	1 or 54	1	1	5	1	1	positive	positive (17/21)	R	R (12/21)	R	R (17/21)
13	P01-04	82 (incl. 8 HEIs)	14	*A*. *baumannii*	1(4)	CC1	1	1	1	1	5	1	1	positive	positive (78/82)	R	R	R	R (75/82)
14	P37-04	1	1	*A*. *baumannii*	1(1)	CC1	1	1	1	1	5	1	1	positive	positive	R	R	R	R
15	P37-05	2	1	*A*. *baumannii*	1(1)	CC1	1	1	1	1	5	1	1	positive	positive	R	R	R	R
16a	P01-07	1	1	*A*. *baumannii*	1	CC1	1	1	1	1	5	1	1	positive	positive	R	R	R	R
16b	P06-05	1	1	*A*. *baumannii*	1	CC1	1	1	1	1	5	1	1	positive	positive	R	R	R	R
17	P44-01	4	1	*A*. *nosocomialis*	313(1)		53	26	47	14	27	16	47	negative	negative	R	R	R	R (2/4)
18	P44-02	2	1	*A*. *nosocomialis*	313(1)		53	26	47	14	27	16	47	negative	negative	R	R	R	R
19	**LUH 05988 (*A*. *nosocomialis*)**			*A*. *nosocomialis*										negative	negative	S	S	S	S
20	P33-01	1	1	*A*. *nosocomialis*										negative	negative	R	S	S	S
21	P36-01	1	1	Close to *A*. *pittii*	312(1)		52	55	53	16	55	36	55	negative	negative	R	S	S	S
22	P03-01	2	2	*A*. *pittii*										negative	negative	R	S	S	S
23	P20-01	2	2	*A*. *pittii*										negative	negative	R	S	S	S
24	**LUH 09695 (*A*. *pittii*)**			*A*. *pittii*										negative	negative	R	S	S	S
25	P43-01	1	1	*A*. *pittii*										negative	negative	R	S	S	S
26	P11-01	2	2	*A*. *baumannii*	193(1)		3	1	7	1	7	2	4	positive	negative	R	S	S	S
27	P45-01	1	1	*A*. *pittii*										negative	negative	R	S	S	S
28	P31-01	2	2	*A*. *pittii*										negative	negative	R	S	S	S
29	P32-01	1	1	*A*. *calcoaceticus*										negative	negative	R	S	S	S
30	P42-01	2	1	*A*. *pittii*										positive	negative	R	S	S	S
31	P25-01	2	2	*A*. *pittii*										negative	negative	S	S	S	S
32	P48-01	1	1	*A*. *pittii*										negative	negative	R	S	S	S
33	P38-01	1	1	*A*. *pittii*										negative	negative	R	S	S	S
34	P39-01	1	1	*A*. *pittii*										negative	negative	R	S	S	S

CC, clonal complex; HEIs, hospital environmental isolates; R, resistant; S, susceptible; ST, sequence type.

**Fig 2:**
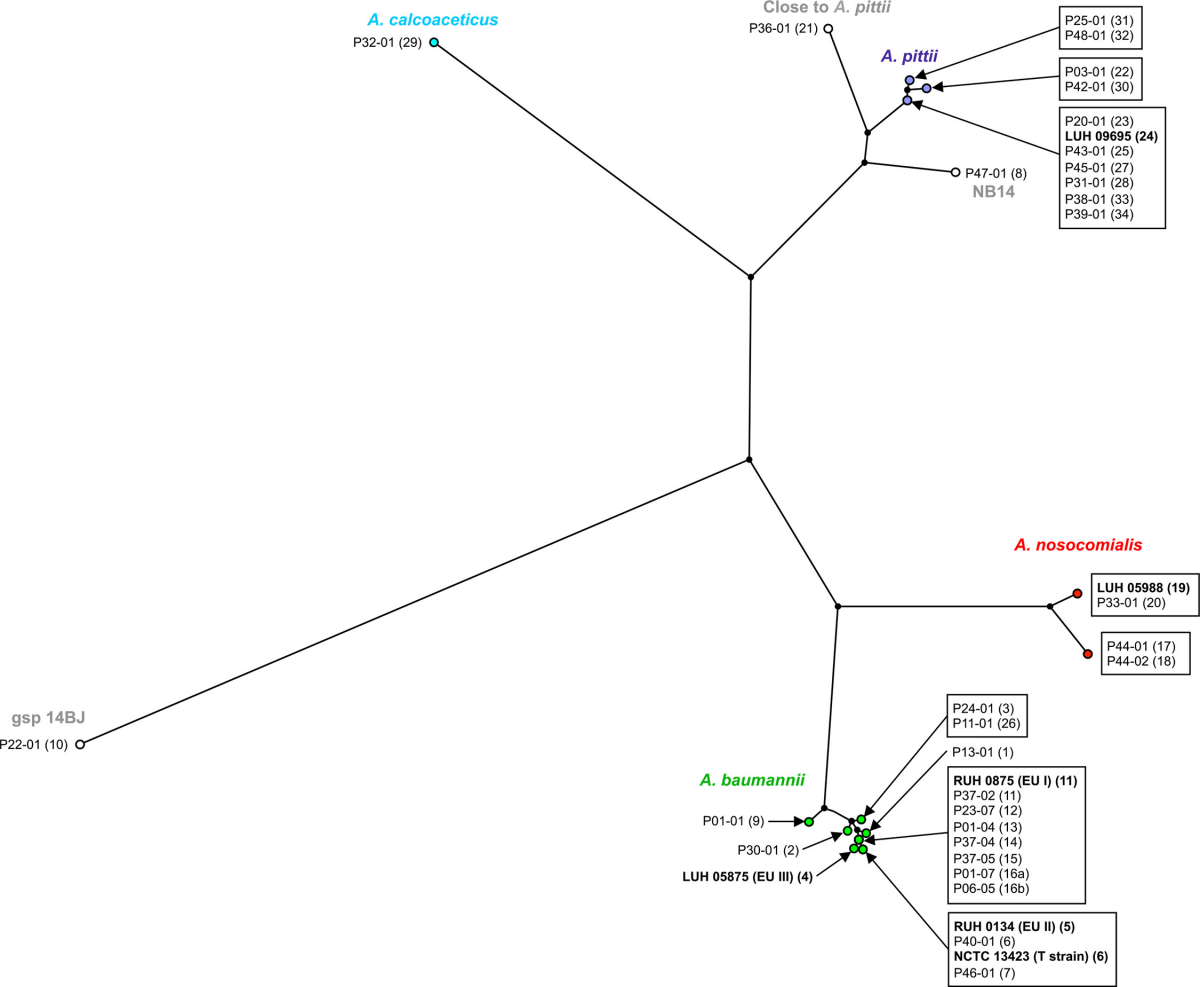
Unrooted neighbor-joining tree representing phylogenetic relationships between the 31 rep-PCR type strains and 6 reference strains. The tree was derived from partial *rpoB* gene sequences. Different colors indicate distinct species within the *Acinetobacter* genus.

One (type) isolate of each rep-type was identified to species by *rpoB* sequence analysis. It was assumed by inductive generalization that isolates of the same rep-type belonged to the same species. Thus, the analysis of the partial *rpoB* gene sequence of type strains allowed for the allocation of 14 of the 31 rep-PCR genotypes to *A*. *baumannii* (i.e. by inductive generalization 131/157 isolates, 83.4%). Likewise, 10 rep-types (15 isolates, 9.6%) were identified to *A*. *pittii*, 3 rep-types (7 isolates, 4.5%) to *A*. *nosocomialis* and 4 rep-types, representing 1 isolate each (0.6%), to *A*. *calcoaceticus*, *A*. *courvalinii* (previously named genomic species 14BJ), NB14 and to ‘close to *A*. *pittii*’ (Fig 2). Twenty patients (41.6%) were colonized with ‘non-baumannii’ *Acinetobacter* strains. No patients were colonized with both baumannii and non-baumannii isolates. Fourteen rep-PCR genotypes harbored an OXA-51-like gene (Table 2). Thirteen of these 14 OXA-51-like positive genotypes were identified as *A*. *baumannii* by *rpoB* sequencing; the remaining genotype (Rep30) was identified as *A*. *pittii* (Table 2 and Fig 2). The *rpoB*-based identification of some ‘non-baumannii’ *Acinetobacter* species was confirmed by 16S rRNA gene sequence analysis (data not shown).

**Fig 3:**
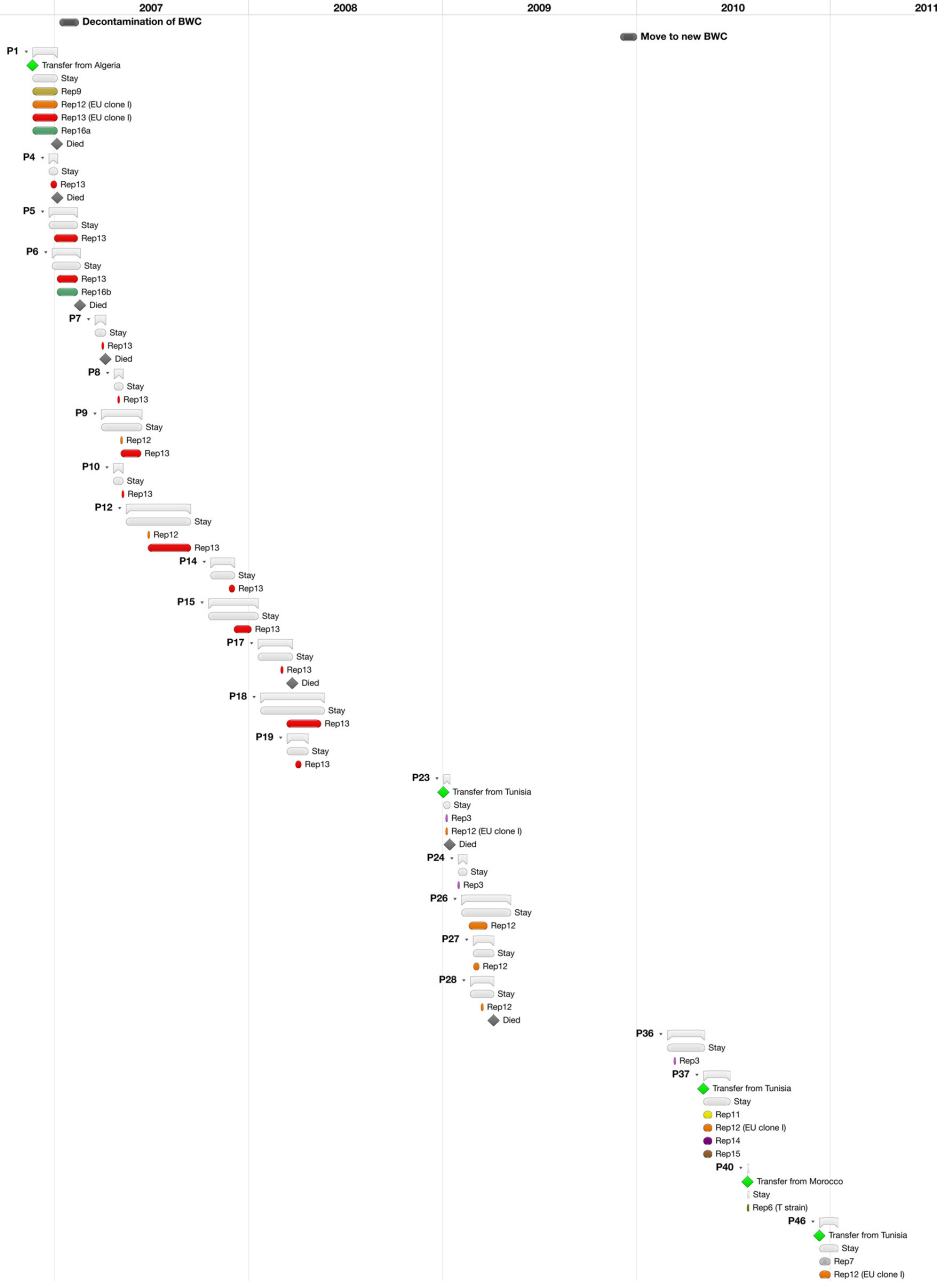
Time course of MDR *A*. *baumannii* colonization/infection in the BWC. Green diamonds indicate the admission of patients from countries known to have a high prevalence of resistance mechanisms. A black diamond marks the death of a patient. Grey bars represent the period of time patients were hospitalized in the BWC, while colored bars indicate the period of time during which they were colonized or infected with a particular MDR *A*. *baumannii* strain (rep-PCR genotype). P, patient.

No less than 12 XDR Acb complex strains were observed. Ten of them were identified to *A*. *baumannii* and 2 to *A*. *nosocomialis*. Rep-PCR profiles indicated that the 2 *A*. *nosocomialis* strains were closely related. All XDR A. *baumannii* strains were non-susceptible to carbapenems and were imported in the BWC through 5 patients, who were injured and subsequently hospitalized in North Africa [Tunisia (*n* = 3), Algeria (*n* = 1) and Morocco (*n* = 1)] and later transferred to the BWC (Table 1). Four non-MDR strains were imported in the BWC when patients were transferred from Belgian hospitals (Table 1). One burn wound patient (Patient 1 in Table 1), who was transferred from Algeria where she had been hospitalized for 8 days, was shown to carry 4 genomically diverse XDR *A*. *baumannii* strains at admission. Two of them, strains Rep12 and 13, were the most prevalent (21 and 82 isolates, from 9 and 14 patients; respectively) in the BWC. Both strains caused consecutive outbreaks, interspersed with short periods of apparent eradication (Fig 3). Patient 1 was co-infected with MDR *P*. *aeruginosa*, ESBL producing *Klebsiella pneumoniae*, *Providencia stuartii* and MRSA. Strain Rep13 appeared to have been introduced once through the transfer of Patient 1and remained present for more than 16 months (from 21 November 2006 to 9 April 2008) (Table 1 and Fig 3), while strain Rep12 was present over the entire study period, apparently as a result of repeated importations into the BWC (through patients 1, 23, 37 and 46) (Table 1 and Fig 3). Both strains spread to the MCU after a few months, but did not spread to other departments such as the consultation and ambulatory services where discharged patients come for follow-up consultation. All hospital environmental isolates belonged to the Rep13 genotype. No non-baumannii *Acinetobacter* outbreaks were observed. Four pairs of patients (P2/3, P20/21, P25/29 and P31/34) were colonized by the same *A*. *pittii* rep-PCR type (each pair with a different rep-type) (Table 1). Patients P2 and P3 were hospitalized at the same time in the same ward, and underwent the same specific treatment (skin expansion reconstruction), suggesting that cross-contamination may have occurred. Pediatric patients P20 and P21 stayed in the same ward, but during different periods of time. They did share two specific clinical interventions (adapted pediatric analgesia and application of a biosynthetic wound dressing). Pairs P25/29 and P31/34 resided in the same ward, but at different times, and shared no specific interventions or equipment.

MLST was used to investigate the prevalence of particular *A*. *baumannii* MLST types (e. g. EU clones I, II and III) in the BWC. Among the confirmed *A*. *baumannii* strains, 9 sequence types (STs) and 3 clonal complexes (CCs), including CC1 (EU clone I), CC2 (EU clone II), and CC10 were observed. MLST revealed that the major epidemic strains in the BWC, Rep12 and 13, were closely related to the EU (or WW) clone I. One burn wound patient (Patient 40), who was transferred from a Moroccan hospital, was shown to carry an XDR representative of the epidemic *A*. *baumannii* T strain, which is related to EU clone II. This strain type has been involved in outbreaks in the United Kingdom and the United States and was associated with repatriated casualties of the Iraq conflict [31]. In our study, the T strain, as well as the remaining 28 minor or sporadic genotypes, did not cause noteworthy outbreaks in the BWC.

Eight *A*. *baumannii* strains were *bla*_OXA-23-like_ positive and carbapenem resistant (Table 2). Two *A*. *nosocomialis* and one *A*. *baumannii* strain showed carbapenem resistance without detection of *bla*_OXA-23-like_. Most isolates of the two major epidemic strains in the BWC (Rep12 and 13) were *bla*_OXA-23-like_ positive (Table 2). A more detailed analysis revealed the presence of an AmpC β-lactamase gene and an IS*Aba1* insertion sequence, upstream of *bla*_AmpC_, but not of *bla*_OXA-23-like_, in both outbreak strains and of a TEM-1-like β-lactamase gene in isolates of the Rep13 genotype (data not shown). No genes coding for additional OXA-carbapenemases (23, 24 and 58-like), β-lactamases or integron sequences were detected (data not shown) in either of the strains.

### Interventions

Identification of contaminated (bed-side) equipment as potential sources of *Acinetobacter* transmission led to the implementation of enhanced hospital environmental decontamination procedures. Unfortunately, even specialized decontamination using vaporized hydrogen peroxide, which necessitated a temporary closure of the BWC (from January 11 to February 15, 2007), did not result in noticeable changes in Acb complex colonization (Fig 3). *Acinetobacter* (cross-)colonization remained an important issue and the major epidemic *A*. *baumanni* strains (Rep12 and 13) were still very much present in the BWC after decontamination (Table 1 and Fig 3). The move to a new burn unit did not result in a considerable decrease of the incidence density of *Acinetobacter* colonization for which colonization prevalence rates were 3.45% (13 of 377 patients) and 2.95% (11 of 373 patients) in the 12 months before and after the move, respectively. However, whereas the admission of two patients colonized with representatives of the epidemic EU clone I (Patients 1 and 23 in Table 1) had led to outbreaks in the ‘old’ BWC, in the new BWC, the admission of two similar patients, also colonized with representatives of EU clone I (Patients 37 and 46) and one patient (P40) carrying a representative of the epidemic T strain (strain P40-01 and the T strain have nearly identical rep-PCR profiles) did not result in outbreaks (Table 1 and Fig 3). Although we are not able to provide statistical data to support this hypothesis, it seems that the stricter infection control measures, made possible by adapted hospital architecture and the introduction of surveillance methods, resulted in the successful containment of these renown epidemic strains.

### Clinical impact

Eight (16.7%) of the 48 Acb complex infected (n = 3) and colonized (n = 5) patients died, including the index patient (Patient 1). All were colonized or infected with *A*. *baumannii* (Table 1), including 7 with CRAB. With the exception of patients 1 and 28, deaths occurred in debilitated middle-aged to elderly patients with a Baux score (comparative indicator of burn severity expressed as: age + %TBSA burned) ranging from 99 to 118 (Table 1). Deaths could not directly be attributed to *A*. *baumannii* infection or colonization. Three CRAB colonized/infected (at the time of death) patients (Patients 1, 4 and 6 in Table 1) died of multiple organ failure related to sepsis (confirmed by positive blood culture) with *P*. *aeruginosa* (unrelated strains) and one from MRSA sepsis (Patient 7). Four patients, who were once colonized with *A*. *baumannii*, but were no longer at time of death, died of causes related to a heart condition (Patient 13), *Serratia marcescens* sepsis (confirmed by positive blood culture) (Patient 28), blood culture negative sepsis (Patient 17) and ventilator-associated pneumonia (VAP) with *P*. *aeruginosa* (Patient 23) (Table 1). One patient (Patient 27, Table 1) survived a culture-confirmed XDR *A*. *baumannii* bloodstream infection. It is, however, plausible to assume that prolonged *A*. *baumannii* infection and colonization represented a contributing factor in the death of some of these patients.

## Discussion

The first XDR *Acinetobacter* outbreak in our BWC, in 2006, triggered an in depth epidemiological investigation of *Acinetobacter* colonization and infection. Phenotypic identification tests such as the VITEK 2 system are insufficient for the identification of *Acinetobacter* isolates to the species or strain level [41]. Definitive species identification by partial *rpoB* sequence analysis of 157 selected Acb complex isolates (identified by VITEK 2) showed that most BWC Acb complex isolates were *A*. *baumannii*. Yet, no less than 20 patients were colonized with ‘non-baumannii’ *Acinetobacter* strains, including 10 strains of *A*. *pittii* and 3 of *A*. *nosocomialis*, both of which species are closely related to *A*. *baumannii*. No spread of ‘non-baumannii’ *Acinetobacter* strains was observed.

Analogous to previous observations of *P*. *aeruginosa* [42], incidence of Acb complex colonization was proportional to the extent of the burn wound, the age of the patient, and the duration of the stay in the BWC. Nosocomially acquired colonization was demonstrated in 83% of *P*. *aeruginosa* colonized patients and in 77.1% of *Acinetobacter* colonized patients. Although *Acinetobacter* species have emerged as nosocomial pathogens from the 1970s onward [43], the *Acinetobacter* colonization rate observed in our BWC from 2006 to 2011 (3.3%) is still considerably lower than that of *P*. *aeruginosa* (16%) in the period 1997 to 1998 [42]. In contrast, a 6-year (2003 to 2008) antibiotic susceptibility records review at the US Army Institute of Surgical Research Burn Center revealed *A*. *baumannii* as the most prevalent organism (22% of isolates), followed by *P*. *aeruginosa* (20% of isolates) [29]. This difference is most likely due to the fact that, in contrast to the US Army burn center, the BWC of the Belgian Army only sporadically repatriated injured military personnel from areas with a high prevalence of MDR organisms. The average time from admission to Acb complex colonization was 14.7 days (range 0–45 days) in our BWC, while in US soldiers wounded in Iraq (2003 to 2005), the median time from injury to identification of Acb complex infection was 6 days (range 3–12 days) [20].

Strain identification to monitor particular strains was done by rep-typing. The discriminatory capacity of rep-PCR is almost as high as other genomic fingerprinting methods used for *Acinetobacter* typing, including fluorescent amplified fragment-length polymorphism (f-AFLP)–a method by which restriction fragments are selectively fluorescently labeled, amplified and electrophoretically separated–and pulsed field gel electrophoresis (PFGE) genomic fingerprinting [32, 44–46]. We opted for the DiversiLab system, a commercially adapted semi-automated rep-PCR system, because of its reproducibility, ease of use and high throughput [47–49]. Thirty-one rep-PCR types/clusters including single strains were distinguished among the 157 isolates from the BWC (Fig 1, Table 2), 12 of them containing XDR isolates. The two predominant XDR *A*. *baumannii* clones in our BWC (Rep12 and 13), causing repeated outbreaks involving 20 patients, were shown to be related to the EU (or WW) clone I, as confirmed by MLST. MLST was used to allocate rep-PCR genotypes to known epidemic clones of *A*. *baumannii* and is an important tool in the study of the population structure and genetic diversity of *A*. *baumannii* [31]. An XDR representative of the epidemic T strain, a highly successful outbreak strain of *A*. *baumannii*, associated with soldiers returning to the United States or United Kingdom from Iraq [31], was imported into the BWC through a patient transferred from a Moroccan hospital. Neither the T strain nor other rep-types had spread in the BWC (some found in multiple patients had no time-space overlap).

The index patient of the first outbreak carried 4 genomically distinct XDR *A*. *baumannii* strains at admission, including the two variants of EU/WW clone I (Rep 12 and 13), indicating that this patient was submitted from a hospital in which these organisms were likely endemic. Recently, Halachev *et al*. [50] also observed that one patient could be infected with more than one species or strain of *Acinetobacter* and could carry a multitude of variants of an outbreak strain. Patients carrying *Acinetobacter* were most often infected with other ESKAPE pathogens such as *P*. *aeruginosa*. During the study period, three additional representatives of Rep12 were likely imported in the BWC through the transfer of three patients from North Africa. In total, 10 XDR *A*. *baumannii* strains were introduced in the BWC through patients injured and hospitalized in North Africa in the period November 2006 to December 2010. The Brussels BWC was thus shown to suffer from consecutive clonal CRAB outbreaks, likely resulting from consecutive importations of outbreak strains. Of note, it can be difficult in regions with complex endemic situations to decide whether particular strains have persisted in a hospital or are reintroduced from other hospitals.

Carbapenem resistance in *A*. *baumannii* is most often mediated by class D oxacillinases (OXAs) [51], combined with low outer membrane permeability [52], and sporadically by metallo-β-lactamases [53]. Carbapenem hydrolysis by OXAs is considered to be too low to confer carbapenem resistance, but association with insertion elements has been shown to increase the expression of these carbapenemases in some cases [54–56]. Two types of OXAs were observed within *Acinetobacter*: intrinsic (chromosomal) OXAs and acquired OXAs. The first intrinsic *A*. *baumannii* OXA carbapenemase gene was detected in 2005 and was named *bla*_OXA-51_ [57]. Since then, many closely related variants were reported [58, 59], which today are referred to as ‘*bla*_OXA-51-like_’. Carbapenem non-susceptibility was only associated with intrinsic *bla*_OXA-51-like_ when the insertion sequence IS*Aba1* was present upstream. The *bla*_OXA-51_ gene has been proposed to be a taxonomic marker for *A*. *baumannii* [36]. However, this has been challenged by the finding of the gene in *A*. *nosocomialis* [60], while we found *bla*_OXA-51-like_ in an *A*. *pittii* clinical isolate. The detection of *bla*_OXA-51-like_ is thus on its own not reliable for the identification of *A*. *baumannii* [61]. Three subgroups of acquired OXAs have been described so far: OXA-23-like, OXA-24/40-like and OXA-58-like [13]. Most isolates of the two major epidemic strains of the BWC, Rep12 and 13 representatives of EU clone I, were *bla*_OXA-23-like_ positive and carbapenem resistant. MDR OXA-23 producing *A*. *baumannii* clones have emerged to cause nosocomial outbreaks worldwide [62–69]. The first CRAB outbreak in our BWC followed the transfer of a patient, with two OXA-23 producing *A*. *baumanni* strains, from North Algeria. CRAB strains have become an increasing cause of concern in Algeria, with OXA-23 producing *A*. *baumannii* being endemic in the north [70]. Both epidemic EU I clones also carried an appropriately oriented IS*Aba*-*ampC* configuration, which was recently shown to increase *ampC* expression, conferring third-generation cephalosporin resistance in *A*. *baumannii* [71]. Rep12 and 13 isolates were indeed resistant to Ceftazidime, the most frequently used third-generation cephalosporin in our BWC during the study period. In addition, strain Rep13 also carried a TEM-1-like β-lactamase gene. TEM-type β-lactamases are most often found in *Escherichia coli* and *K*. *pneumonia*. It has been suggested that TEM-1 represents a clinically relevant mechanism of sulbactam (a β-lactamase inhibitor) resistance in *A*. *baumannii* [72].

There is a continuing controversy regarding the attributable mortality of *A*. *baumannii* infections [73]. From a review of case-control studies it was concluded that inappropriate antibiotic treatment of *A*. *baumannii* infections was associated with increased mortality [16]. All *Acinetobacter* strains we isolated were sensitive to colistin, which allowed for colistin (usually combined with rifampicin) treatments of CRAB infected patients. Since only two patients were treated with colistin monotherapy (without addition of rifampicin), it is impossible to know if the addition of rifampicin implied a clinical benefit. In a recent study [74], a significant increase of microbiologic eradication rate was observed when colistin was combined with rifampicin, but no difference was observed for infection-related death and length of hospitalization. Also taking into account the emergence of rifampicin resistance [75], this might imply that rifampicin should not be added to colistin in routine clinical practice [74]. Except a moderate renal impairment in one patient, no major adverse events related to colistin treatment were observed. From the analysis of 5 studies published between 1999 and 2006 in which patients with pneumonia due to *P*. *aeruginosa* or *A*. *baumannii* were treated with intravenous colistin, it was reported that *de novo* nephrotoxicity occured in 8–36% of patients, while neurotoxicity, which was commonly described in the old colistin era, was rarely observed [76].

Sample sizes are too small to perform adequate statistical analysis of the clinical impact of *Acinetobacter* infection and colonization. Eight (16.7%) out of 48 *Acinetobacter* colonized or infected patients died. Although no autopsies were performed, there were no indications that *Acinetobacter* infection and colonization was associated with increased attributable mortality, suggesting that the implemented antibiotic treatment was overall successful. Six of the eight deceased patients had Baux scores ranging from 99 to 118, scores at which predicted mortality is high. A 27-year retrospective study, including 11,109 burn wound patients, showed that the Baux score at which predicted mortality reached 50% was 109.6 in the 2000 to 2008 cohort [77]. This finding is congruent with a previous study that found that although MDR Acb is a common cause of nosocomial infection in the burn patient population, it does not independently affect mortality [78]. In comparison, 8 out of 70 (11.4%) *P*. *aeruginosa* colonized patients died and for 3 of them, death was directly attributed to *P*. *aeruginosa* infection [36]. ‘Non-baumannii’ *Acinetobacter* species, and especially *A*. *nosocomialis* and *A*. *pittii*, are increasingly recognized as significant causes of nosocomial infection [2, 79–83]. The mortality rate for patients infected with ‘non-baumanii’ *Acinetobacter* strains is considered to be lower than for *A*. *baumannii* [81, 83]. Poor outcome seemed to be associated with MDR strains rather than with *A*. *baumannii per se* [81]. With the exception of two closely related XDR *A*. *nosocomialis* strains, ‘non-baumanni’ *Acinetobacter* strains were relatively susceptible to antibiotics and as a consequence the initial (empiric) antimicrobial therapy was appropriate. None of the ‘non-baumannii’ *Acinetobacter* colonized or infected patients died. The mean length of colonization/infection was only 4.9 days for non-baumannii *Acinetobacter* as compared to 19.1 days for *A*. *baumannii* strains (Table 1). In contrast to *A*. *baumannii* strains, and more specifically epidemic strains, which were shown to colonize/infect multiple sites of the burned patients, non-baumannii *Acinetobacter* strains were mostly isolated from wounds. These differences are possibly due to the more pronounced antibiotic resistance of *A*. *baumannii* strains.

The natural reservoir of *A*. *baumannii* is still unknown and the human body does not seem to be a natural habitat for *A*. *baumannii*. In 1999, Berlau and coworkers determined the distribution of 19 genospecies of *Acinetobacter* on human skin (forehead, forearm and toe webs). Over 40% of 192 healthy volunteers carried *Acinetobacter* spp. at one or more body sites, with *Acinetobacter lwoffii* as the most common (61%) colonizer, and the Acb complex was found in only one individual [84]. Skin or nasal carriage of Acb complex was also not detected among healthy US Army soldiers [85, 86]. However, certain MDR epidemic strains can survive in the hospital setting. Susceptible, critically ill patients and their environment become a niche or reservoir from which transmission to other patients and environment occurs [7]. Contaminated healthcare workers may be vectors for transmission [87, 88], although a screening of the BWC’s healthcare personnel during an outbreak did not provide evidence for this. The epidemic Rep13 strain was recovered from the patients’ environment on several occasions. Likely, this contamination was secondary to patient carriage. *A*. *baumannii* strains have the ability to survive for a long time on dry surfaces under simulated hospital conditions, and this irrespective of their sporadic or epidemic occurrence [89]. The apparent absence of *Acinetobacter* in healthcare workers, the recovery of the major epidemic *A*. *baumannii* strain from the direct environment of colonized patients and the continuous admittance and presence of colonized patients in the BWC suggest that the epidemic strains entered the BWC through colonized patients and that prolonged carriage and hospital environmental contamination were responsible for the spread to other susceptible patients, possibly through contaminated equipment.

Despite systematic screening on admission, draconian hospital hygiene measures based on intensive surveillance methods and a temporary closure of the BWC for specialized decontamination, two imported OXA-23 producing EU I clones persisted in the BWC, causing recurrent outbreaks. Desiccation tolerance and excessive drug resistance may have contributed to their maintenance in the BWC and their propensity to cause outbreaks. It seems that the move to a new BWC with an adapted architecture finally allowed for the implementation of infection control measures necessary for the containment of these epidemic strains. However, there is a lack of evidence linking hospital design and construction with the prevention of nosocomial infection and it seems that other factors, especially the improper hand hygiene of medical staff, might have greater impact [90].

## Conclusions

This study adds to previous observations that hospitals are vulnerable to importation of MDR *A*. *baumannii* when patients are transferred from hospitals in countries known to have a high prevalence of resistance mechanisms. It is no surprise that the military BWC in Brussels was confronted with MDR *A*. *baumannii* outbreaks. Patients with extensive burn wounds are particularly at risk for infection and military hospitals often admit patients that were repatriated from regions where they were exposed to endemic or emerging MDR pathogens such as OXA-23 producing *A*. *baumannii*. This study also underlines the importance of systematic screening upon admission and early application of strict barrier precautions for any transferred patient, whatever the country of origin [91–93]. Rapid automated identification and typing methods (e.g. VITEK and DiversiLab systems) are essential tools in the epidemiological investigation and surveillance of (*Acinetobacter*) outbreaks. The timely identification of highly related or indistinguishable isolates, suggesting transmission from the environment, health care workers or patients, may lead to successful interventions to control major MDR pathogens such as *A*. *baumannii*. More elaborate and robust genetic identification and typing methods such as MLST and *rpoB* gene sequence analysis are of scientific importance. They can be used to assign strains to renown epidemic clones or to perform taxonomic or population structure studies. The setup of bacterial culture collections and fingerprint databases with relevant clinical isolates and reference strains is recommended. The role of non-baumannii *Acinetobacter* in human infections is increasingly reported thanks to these new molecular biology techniques that allow correct identification of bacteria at the species level. Although numerous patients were shown to be colonized by non-baumannii *Acinetobacter* strains, these bacteria were not associated with infections or outbreaks in our BWC. Of concern, two closely related *A*. *nosocomialis* strains were XDR with resistance to carbapenems. Finally, this study supports the assumption that *Acinetobacter* infection and colonization is not associated with increased attributable mortality, provided that an appropriate antibiotic (e.g. colistin/rifampicin) treatment is timely initiated.

## References

[1] Nemec A, Radolfova-Krizova L, Maixnerova M, Vrestiakova E, Jezek P, Sedo O. Taxonomy of haemolytic and/or proteolytic strains of the genus *Acinetobacter* with the proposal of *Acinetobacter courvalinii* sp. nov. (genomic species 14 sensu Bouvet & Jeanjean), *Acinetobacter dispersus* sp. nov. (genomic species 17), *Acinetobacter modestus* sp. nov., *Acinetobacter proteolyticus* sp. nov. and *Acinetobacter vivianii* sp. nov. Int J Syst Evol Microbiol. 2016;66: 1673–1685. 10.1099/ijsem.0.000932 26822020

[2] Nemec A, Krizova L, Maixnerova M, van der Reijden TJ, Deschaght P, Passet V et al Genotypic and phenotypic characterization of the *Acinetobacter calcoaceticus*-*Acinetobacter baumannii* complex with the proposal of *Acinetobacter pittii* sp. nov. (formerly *Acinetobacter* genomic species 3) and *Acinetobacter nosocomialis* sp. nov. (formerly *Acinetobacter* genomic species 13TU). Res Microbiol. 2011;162: 393–404. 10.1016/j.resmic.2011.02.006 21320596

[3] Nemec A, Krizova L, Maixnerova M, Sedo O, Brisse S, Higgins PG. *Acinetobacter seifertii* sp. nov., a member of the *Acinetobacter calcoaceticus*-*Acinetobacter baumannii* complex isolated from human clinical specimens. Int J Syst Evol Microbiol. 2015;65: 934–942. 10.1099/ijs.0.000043 25563912

[4] Gerner-Smidt P, Tjernberg I. *Acinetobacter* in Denmark: II. Molecular studies of the *Acinetobacter calcoaceticus*-*Acinetobacter baumannii* complex. APMIS. 1993;101: 826–832. 8286091

[5] Espinal P, Mosqueda N, Telli M, van der Reijden T, Rolo D, Fernández-Orth D et al Identification of NDM-1 in a putatively novel *Acinetobacter* species ("NB14") closely related to *Acinetobacter pittii*. Antimicrob Agents Chemother. 2015;59: 6657–6660. 10.1128/AAC.01455-15 26259796 PMC4576105

[6] Adegoke AA, Mvuyo T, Okoh AI. Ubiquitous *Acinetobacter* species as beneficial commensals but gradually being emboldened with antibiotic resistance genes. J Basic Microbiol. 2012;52: 620–627. 10.1002/jobm.201100323 22359150

[7] Dijkshoorn L, Nemec A, Seifert H. An increasing threat in hospitals: multidrug-resistant *Acinetobacter baumannii*. Nat Rev Microbiol. 2007;5: 939–951. 18007677 10.1038/nrmicro1789

[8] Rice LB. Federal funding for the study of antimicrobial resistance in nosocomial pathogens: no ESKAPE. J Infect Dis. 2008;197: 1079–1081. 10.1086/533452 18419525

[9] Antunes LC, Visca P, Towner KJ. *Acinetobacter baumannii*: evolution of a global pathogen. Pathog Dis. 2014;71: 292–301. 10.1111/2049-632X.12125 24376225

[10] Dijkshoorn L, Aucken H, Gerner-Smidt P, Janssen P, Kaufmann ME, Garaizar J et al Comparison of outbreak and nonoutbreak *Acinetobacter baumannii* strains by genotypic and phenotypic methods. J Clin Microbiol. 1996;34: 1519–1525. 8735109 10.1128/jcm.34.6.1519-1525.1996 PMC229053

[11] van Dessel H, Dijkshoorn L, van der Reijden T, Bakker N, Paauw A, van den Broek P et al Identification of a new geographically widespread multiresistant *Acinetobacter baumannii* clone from European hospitals. Res Microbiol. 2004;155: 105–112. 14990262 10.1016/j.resmic.2003.10.003

[12] Diancourt L, Passet V, Nemec A, Dijkshoorn L, Brisse S. The population structure of *Acinetobacter baumannii*: expanding multiresistant clones from an ancestral susceptible genetic pool. PLoS One. 2010;5: e10034 10.1371/journal.pone.0010034 20383326 PMC2850921

[13] Higgins PG, Dammhayn C, Hackel M, Seifert H. Global spread of carbapenem-resistant *Acinetobacter baumannii*. J Antimicrob Chemother. 2010;65: 233–238. 10.1093/jac/dkp428 19996144

[14] Higgins PG, Dammhayn C, Hackel M, Seifert H. Erratum in: J Antimicrob Chemother. 2010;65: 1317. 10.1093/jac/dkp428 19996144

[15] Zarrilli R, Pournaras S, Giannouli M, Tsakris A. Global evolution of multidrug-resistant *Acinetobacter baumannii* clonal lineages. Int J Antimicrob Agents. 2013;41: 11–19. 10.1016/j.ijantimicag.2012.09.008 23127486

[16] Falagas ME, Rafailidis PI. Attributable mortality of *Acinetobacter baumannii*: no longer a controversial issue. Crit Care. 2007;11: 134 17543135 10.1186/cc5911 PMC2206403

[17] Urban C, Mariano N, Rahal JJ, Tay E, Ponio C, Koprivnjak T et al Polymyxin B-resistant *Acinetobacter baumannii* clinical isolate susceptible to recombinant BPI and cecropin P1. Antimicrob Agents Chemother. 2001;45: 994–995. 16557680 10.1128/AAC.45.3.994-995.2001 PMC90414

[18] Snitkin ES, Zelazny AM, Montero CI, Stock F, Mijares L; NISC Comparative Sequence Program, Murray PR, Segre JA. Genome-wide recombination drives diversification of epidemic strains of *Acinetobacter baumannii*. Proc Natl Acad Sci U S A. 2011;108: 13758–13763. 10.1073/pnas.1104404108 21825119 PMC3158218

[19] Chusri S, Chongsuvivatwong V, Rivera JI, Silpapojakul K, Singkhamanan K, McNeil E et al Clinical outcomes of hospital-acquired infection with *Acinetobacter nosocomialis* and *Acinetobacter pittii*. Antimicrob Agents Chemother. 2014;58: 4172–4179. 10.1128/AAC.02992-14 24820079 PMC4068534

[20] Davis KA, Moran KA, McAllister CK, Gray PJ. Multidrug-resistant *Acinetobacter* extremity infections in soldiers. Emerg Infect Dis. 2005;11: 1218–1224. 16102310 10.3201/1108.050103 PMC3320488

[21] Jones A, Morgan D, Walsh A, Turton J, Livermore D, Pitt T et al Importation of multidrug-resistant *Acinetobacter* spp infections with casualties from Iraq. Lancet Infect Dis. 2006;6: 317–318. 16728314 10.1016/S1473-3099(06)70471-6

[22] Scott P, Deye G, Srinivasan A, Murray C, Moran K, Hulten E et al An outbreak of multidrug-resistant *Acinetobacter baumannii*-*calcoaceticus* complex infection in the US military health care system associated with military operations in Iraq. Clin Infect Dis. 2007;44: 1577–1584. 17516401 10.1086/518170

[23] Sebeny PJ, Riddle MS, Petersen K. *Acinetobacter baumannii* skin and soft-tissue infection associated with war trauma. Clin Infect Dis. 2008;47: 444–449. 10.1086/590568 18611157

[24] Keen EF 3rd, Murray CK, Robinson BJ, Hospenthal DR, Co EM, Aldous WK. Changes in the incidences of multidrug-resistant and extensively drug-resistant organisms isolated in a military medical center. Infect. Control Hosp Epidemiol. 2010;31: 728–732. 10.1086/653617 20500036

[25] Petersen K, Cannegieter SC, van der Reijden TJ, van Strijen B, You DM, Babel BS et al Diversity and clinical impact of *Acinetobacter baumannii* colonization and infection at a military medical center. J Clin Microbiol. 2011;49: 159–166. 10.1128/JCM.00766-10 21084513 PMC3020478

[26] O'Shea MK. *Acinetobacter* in modern warfare. Int J Antimicrob Agents. 2012;39: 363–375. 10.1016/j.ijantimicag.2012.01.018 22459899

[27] Church D, Elsayed S, Reid O, Winston B, Lindsay R. Burn wound infections. Clin Microbiol Rev. 2006;19: 403–434. 16614255 10.1128/CMR.19.2.403-434.2006 PMC1471990

[28] Trottier V, Segura PG, Namias N, King D, Pizano LR, Schulman CI. Outcomes of *Acinetobacter baumannii* infection in critically ill burned patients. J Burn Care Res. 2007;28: 248–254. 17351441 10.1097/BCR.0B013E318031A20F

[29] Keen EF 3rd, Robinson BJ, Hospenthal DR, Aldous WK, Wolf SE, Chung KK et al Prevalence of multidrug-resistant organisms recovered at a military burn center. Burns. 2010;36: 819–825. 10.1016/j.burns.2009.10.013 20080354

[30] Magiorakos AP, Srinivasan A, Carey RB, Carmeli Y, Falagas ME, Giske CG et al Multidrug-resistant, extensively drug-resistant and pandrug-resistant bacteria: an international expert proposal for interim standard definitions for acquired resistance. Clin Microbiol Infect. 2012;18: 268–281. 10.1111/j.1469-0691.2011.03570.x 21793988

[31] Turton JF, Kaufmann ME, Gill MJ, Pike R, Scott PT, Fishbain J et al Comparison of *Acinetobacter baumannii* isolates from the United Kingdom and the United States that were associated with repatriated casualties of the Iraq conflict. J Clin Microbiol. 2006;44: 2630–2634. 16825400 10.1128/JCM.00547-06 PMC1489513

[32] Saeed S, Fakih MG, Riederer K, Shah AR, Khatib R. Interinstitutional and intrainstitutional transmission of a strain of *Acinetobacter baumannii* detected by molecular analysis: comparison of pulsed-field gel electrophoresis and repetitive sequence-based polymerase chain reaction. Infect Control Hosp Epidemiol. 2006;27: 981–983. 16941328 10.1086/507286

[33] Higgins PG, Janssen K, Fresen MM, Wisplinghoff H, Seifert H. Molecular epidemiology of *Acinetobacter baumannii* bloodstream isolates obtained in the United States from 1995 to 2004 using rep-PCR and multilocus sequence typing. J Clin Microbiol. 2012;50: 3493–3500. 10.1128/JCM.01759-12 22895032 PMC3486219

[34] La Scola B, Gundi VA, Khamis A, Raoult D. Sequencing of the *rpoB* gene and flanking spacers for molecular identification of *Acinetobacter* species. J Clin Microbiol. 2006;44: 827–832. 16517861 10.1128/JCM.44.3.827-832.2006 PMC1393131

[35] Turton JF, Kaufmann ME, Glover J, Coelho JM, Warner M, Pike R et al Detection and typing of integrons in epidemic strains of *Acinetobacter baumannii* found in the United Kingdom. J Clin Microbiol. 2005;43: 3074–3082. 16000417 10.1128/JCM.43.7.3074-3082.2005 PMC1169174

[36] Turton JF, Woodford N, Glover J, Yarde S, Kaufmann ME, Pitt TL. Identification of *Acinetobacter baumannii* by detection of the *bla*_OXA-51-like_ carbapenemase gene intrinsic to this species. J Clin Microbiol. 2006;44: 2974–2976. 16891520 10.1128/JCM.01021-06 PMC1594603

[37] Bogaerts P, Rezende de Castro R, de Mendonça R, Huang TD, Denis O, Glupczynski Y. Validation of carbapenemase and extended-spectrum β-lactamase multiplex endpoint PCR assays according to ISO 15189. J Antimicrob Chemother. 2013;68: 1576–1582. 10.1093/jac/dkt065 23508620

[38] Bogaerts P, Cuzon G, Naas T, Bauraing C, Deplano A, Lissoir B et al Carbapenem-resistant *Acinetobacter baumannii* isolates expressing the *bla*_OXA-23_ gene associated with IS*Aba4* in Belgium. Antimicrob Agents Chemother. 2008;52: 4205–4206. 10.1128/AAC.01121-08 18765689 PMC2573094

[39] Vaneechoutte M, Claeys G, Steyaert S, De Baere T, Peleman R, Verschraegen G. Isolation of *Moraxella canis* from an ulcerated metastatic lymph node. J Clin Microbiol. 2000;38: 3870–3871. 11015424 10.1128/jcm.38.10.3870-3871.2000 PMC87497

[40] Giamarellos-Bourboulis EJ, Xirouchaki E, Giamarellou H. Interactions of colistin and rifampin on multidrug-resistant *Acinetobacter baumannii*. Diagn Microbiol Infect Dis. 2001;40: 117–120. 11502379 10.1016/s0732-8893(01)00258-9

[41] Gerner-Smidt P, Tjernberg I, Ursing J. Reliability of phenotypic tests for identification of *Acinetobacter* species. J Clin Microbiol. 1991;29: 277–282. 2007635 10.1128/jcm.29.2.277-282.1991 PMC269753

[42] Pirnay JP, De Vos D, Cochez C, Bilocq F, Pirson J, Struelens M et al Molecular epidemiology of *Pseudomonas aeruginosa* colonization in a burn unit: persistence of a multidrug-resistant clone and a silver sulfadiazine-resistant clone. J Clin Microbiol. 2003;41: 1192–1202. 12624051 10.1128/JCM.41.3.1192-1202.2003 PMC150314

[43] Towner KJ. *Acinetobacter*: an old friend, but a new enemy. J Hosp Infect. 2009;73: 355–363. 10.1016/j.jhin.2009.03.032 19700220

[44] Vila J, Marcos MA, Jimenez de Anta MT. A comparative study of different PCR-based DNA fingerprinting techniques for typing of the *Acinetobacter calcoaceticus*-*A*. *baumannii* complex. J Med Microbiol. 1996;44: 482–489. 8636966 10.1099/00222615-44-6-482

[45] Snelling AM, Gerner-Smidt P, Hawkey PM, Heritage J, Parnell P, Porter C et al Validation of use of whole-cell repetitive extragenic palindromic sequence-based PCR (REP-PCR) for typing strains belonging to the *Acinetobacter calcoaceticus*-*Acinetobacter baumannii* complex and application of the method to the investigation of a hospital outbreak. J Clin Microbiol. 1996;34: 1193–1202. 8727902 10.1128/jcm.34.5.1193-1202.1996 PMC228981

[46] Fontana C, Favaro M, Minelli S, Bossa MC, Testore GP, Leonardis F et al *Acinetobacter baumannii* in intensive care unit: a novel system to study clonal relationship among the isolates. BMC Infect Dis. 2008;8: 79 10.1186/1471-2334-8-79 18538034 PMC2443154

[47] Healy M, Huong J, Bittner T, Lising M, Frye S, Raza S et al Microbial DNA typing by automated repetitive-sequence-based PCR. J Clin Microbiol. 2005;43: 199–207. 15634972 10.1128/JCM.43.1.199-207.2005 PMC540112

[48] Higgins PG, Hujer AM, Hujer KM, Bonomo RA, Seifert H. Interlaboratory reproducibility of DiversiLab rep-PCR typing and clustering of *Acinetobacter baumannii* isolates. J Med Microbiol. 2012;61: 137–141. 10.1099/jmm.0.036046-0 21903821 PMC3347881

[49] Decker BK, Perez F, Hujer AM, Hujer KM, Hall GS, Jacobs MR et al Longitudinal analysis of the temporal evolution of *Acinetobacter baumannii* strains in Ohio, USA, by using rapid automated typing methods. PLoS One. 2012;7: e33443 10.1371/journal.pone.0033443 22511922 PMC3325217

[50] Halachev MR, Chan JZ, Constantinidou CI, Cumley N, Bradley C, Smith-Banks M et al Genomic epidemiology of a protracted hospital outbreak caused by multidrug-resistant *Acinetobacter baumannii* in Birmingham, England. Genome Med. 2014;6: 70 10.1186/s13073-014-0070-x 25414729 PMC4237759

[51] Donald HM, Scaife W, Amyes SG, Young HK. Sequence analysis of ARI-1, a novel OXA beta-lactamase, responsible for imipenem resistance in *Acinetobacter baumannii* 6B92. Antimicrob Agents Chemother. 2000;44: 196–199. 10602749 10.1128/aac.44.1.196-199.2000 PMC89654

[52] Sato K, Nakae T. Outer membrane permeability of *Acinetobacter calcoaceticus* and its implication in antibiotic resistance. J Antimicrob Chemother. 1991;28: 35–45. 10.1093/jac/28.1.35 1722802

[53] Poirel L, Nordmann P. Carbapenem resistance in *Acinetobacter baumannii*: mechanisms and epidemiology. Clin Microbiol Infect. 2006;12: 826–836. 16882287 10.1111/j.1469-0691.2006.01456.x

[54] Turton JF, Ward ME, Woodford N, Kaufmann ME, Pike R, Livermore DM et al The role of ISAba1 in expression of OXA carbapenemase genes in *Acinetobacter baumannii*. FEMS Microbiol Lett. 2006;258: 72–77. 16630258 10.1111/j.1574-6968.2006.00195.x

[55] Poirel L, Nordmann P. Genetic structures at the origin of acquisition and expression of the carbapenem-hydrolyzing oxacillinase gene *bla*_OXA-58_ in *Acinetobacter baumannii*. Antimicrob Agents Chemother. 2006;50: 1442–1448. 16569863 10.1128/AAC.50.4.1442-1448.2006 PMC1426978

[56] Segal H, Garny S, Elisha BG. Is ISABA-1 customized for *Acinetobacter*? FEMS Microbiol Lett. 2005;243: 425–429. 15686845 10.1016/j.femsle.2005.01.005

[57] Brown S, Young HK, Amyes SG. Characterisation of OXA-51, a novel class D carbapenemase found in genetically unrelated clinical strains of *Acinetobacter baumannii* from Argentina. Clin Microbiol Infect. 2005;11: 15–23. 10.1111/j.1469-0691.2004.01016.x 15649299

[58] Brown S, Amyes SG. The sequences of seven class D beta-lactamases isolated from carbapenem-resistant *Acinetobacter baumannii* from four continents. Clin Microbiol Infect. 2005;11: 326–329. 15760431 10.1111/j.1469-0691.2005.01096.x

[59] Héritier C, Poirel L, Fournier PE, Claverie JM, Raoult D, Nordmann P. Characterization of the naturally occurring oxacillinase of *Acinetobacter baumannii*. Antimicrob Agents Chemother. 2005;49: 4174–4179. 16189095 10.1128/AAC.49.10.4174-4179.2005 PMC1251506

[60] Lee YT, Turton JF, Chen TL, Wu RC, Chang WC, Fung CP et al First identification of *bla*_OXA-51-like_ in non-*baumannii* Acinetobacter spp. J Chemother. 2009;21: 514–520. 19933042 10.1179/joc.2009.21.5.514

[61] Zander E, Higgins PG, Fernández-González A, Seifert H. Detection of intrinsic *bla*_OXA-51-like_ by multiplex PCR on its own is not reliable for the identification of *Acinetobacter baumannii*. Int J Med Microbiol. 2013;303: 88–89. 10.1016/j.ijmm.2012.12.007 23375845

[62] Dalla-Costa LM, Coelho JM, Souza HA, Castro ME, Stier CJ, Bragagnolo KL et al Outbreak of carbapenem-resistant *Acinetobacter baumannii* producing the OXA-23 enzyme in Curitiba, Brazil. J Clin Microbiol. 2003;41: 3403–3406. 12843104 10.1128/JCM.41.7.3403-3406.2003 PMC165295

[63] Kohlenberg A, Brümmer S, Higgins PG, Sohr D, Piening BC, de Grahl C et al Outbreak of carbapenem-resistant *Acinetobacter baumannii* carrying the carbapenemase OXA-23 in a German university medical centre. J Med Microbiol. 2009;58: 1499–1507. 10.1099/jmm.0.012302-0 19589905

[64] Liakopoulos A, Miriagou V, Katsifas EA, Karagouni AD, Daikos GL, Tzouvelekis LS et al Identification of OXA-23-producing *Acinetobacter baumannii* in Greece, 2010 to 2011. Euro Surveill. 2012;17: 20117 22449866

[65] Peymani A, Higgins PG, Nahaei MR, Farajnia S, Seifert H. Characterisation and clonal dissemination of OXA-23-producing *Acinetobacter baumannii* in Tabriz, northwest Iran. Int J Antimicrob Agents. 2012;39: 526–528. 10.1016/j.ijantimicag.2012.02.014 22521767

[66] Diene SM, Fall B, Kempf M, Fenollar F, Sow K, Niang B et al Emergence of the OXA-23 carbapenemase-encoding gene in multidrug-resistant *Acinetobacter baumannii* clinical isolates from the Principal Hospital of Dakar, Senegal. Int J Infect Dis. 2013;17: e209–210. 10.1016/j.ijid.2012.09.007 23084970

[67] Lee Y, Kim YR, Kim J, Park YJ, Song W, Shin JH et al Increasing prevalence of *bla*_OXA-23_-carrying *Acinetobacter baumannii* and the emergence of *bla*_OXA-182_-carrying *Acinetobacter nosocomialis* in Korea. Diagn Microbiol Infect Dis. 2013;77: 160–163. 10.1016/j.diagmicrobio.2013.06.009 23891219

[68] Olaitan AO, Berrazeg M, Fagade OE, Adelowo OO, Alli JA, Rolain JM. Emergence of multidrug-resistant *Acinetobacter baumannii* producing OXA-23 carbapenemase, Nigeria. Int J Infect Dis. 2013;17: e469–470. 10.1016/j.ijid.2012.12.008 23337460

[69] Merino M, Poza M, Roca I, Barba MJ, Sousa MD, Vila J et al Nosocomial outbreak of a multiresistant *Acinetobacter baumannii* expressing OXA-23 carbapenemase in Spain. Microb Drug Resist. 2014;20: 259–263. 10.1089/mdr.2013.0127 24328852

[70] Baba Ahmed-Kazi Tani Z, Arlet G. News of antibiotic resistance among Gram-negative bacilli in Algeria. Pathol Biol (Paris). 2014;62: 169–178. 24819127 10.1016/j.patbio.2014.01.005

[71] Hamidian M, Hall RM. Resistance to third-generation cephalosporins in *Acinetobacter baumannii* due to horizontal transfer of a chromosomal segment containing ISAba1-*ampC*. J Antimicrob Chemother. 2014;69: 2865–2866. 10.1093/jac/dku202 24917581

[72] Krizova L, Poirel L, Nordmann P, Nemec A. TEM-1 β-lactamase as a source of resistance to sulbactam in clinical strains of *Acinetobacter baumannii*. J Antimicrob Chemother. 2013;68: 2786–2791. 10.1093/jac/dkt275 23838947

[73] Fournier PE, Richet H. The epidemiology and control of *Acinetobacter baumannii* in health care facilities. Clin Infect Dis. 2006;42: 692–699. 16447117 10.1086/500202

[74] Durante-Mangoni E, Signoriello G, Andini R, Mattei A, De Cristoforo M, Murino P et al Colistin and rifampicin compared with colistin alone for the treatment of serious infections due to extensively drug-resistant *Acinetobacter baumannii*: a multicenter, randomized clinical trial. Clin Infect Dis. 2013;57: 349–358. 10.1093/cid/cit253 23616495

[75] Giannouli M, Di Popolo A, Durante-Mangoni E, Bernardo M, Cuccurullo S, Amato G et al Molecular epidemiology and mechanisms of rifampicin resistance in *Acinetobacter baumannii* isolates from Italy. Int J Antimicrob Agents. 2012;39: 58–63. 10.1016/j.ijantimicag.2011.09.016 22055530

[76] Linden PK, Paterson DL. Parenteral and inhaled colistin for treatment of ventilator-associated pneumonia. Clin Infect Dis. 2006;43: S89–94. 16894521 10.1086/504485

[77] Roberts G, Lloyd M, Parker M, Martin R, Philp B, Shelley O et al The Baux score is dead. Long live the Baux score: a 27-year retrospective cohort study of mortality at a regional burns service. J Trauma Acute Care Surg. 2012;72: 251–256. 10.1097/TA.0b013e31824052bb 22310134

[78] Albrecht MC, Griffith ME, Murray CK, Chung KK, Horvath EE, Ward JA et al Impact of Acinetobacter infection on the mortality of burn patients. J Am Coll Surg. 2006;203: 546–550. 17000400 10.1016/j.jamcollsurg.2006.06.013

[79] Idzenga D, Schouten MA, van Zanten AR. Outbreak of *Acinetobacter* genomic species 3 in a Dutch intensive care unit. J Hosp Infect. 2006;63: 485–487. 16815591 10.1016/j.jhin.2006.03.014

[80] Turton JF, Shah J, Ozongwu C, Pike R. Incidence of *Acinetobacter* species other than *A*. *baumannii* among clinical isolates of *Acinetobacter*: evidence for emerging species. J Clin Microbiol. 2010;48: 1445–1449. 10.1128/JCM.02467-09 20181894 PMC2849580

[81] Lee YC, Huang YT, Tan CK, Kuo YW, Liao CH, Lee PI et al *Acinetobacter baumannii* and *Acinetobacter* genospecies 13TU and 3 bacteraemia: comparison of clinical features, prognostic factors and outcomes. J Antimicrob Chemother. 2011;66: 1839–1846. 10.1093/jac/dkr200 21653602

[82] Schleicher X, Higgins PG, Wisplinghoff H, Körber-Irrgang B, Kresken M, Seifert H. Molecular epidemiology of *Acinetobacter baumannii* and *Acinetobacter nosocomialis* in Germany over a 5-year period (2005–2009). Clin Microbiol Infect. 2013;19: 737–742. 10.1111/1469-0691.12026 23034071

[83] Wisplinghoff H, Paulus T, Lugenheim M, Stefanik D, Higgins PG, Edmond MB et al Nosocomial bloodstream infections due to *Acinetobacter baumannii*, *Acinetobacter pittii* and *Acinetobacter nosocomialis* in the United States. J Infect. 2012;64: 282–290. 10.1016/j.jinf.2011.12.008 22209744

[84] Berlau J, Aucken H, Malnick H, Pitt T. Distribution of Acinetobacter species on skin of healthy humans. Eur J Clin Microbiol Infect Dis. 1999;18: 179–183. 10357050 10.1007/s100960050254

[85] Griffith ME, Lazarus DR, Mann PB, Boger JA, Hospenthal DR, Murray CK. Acinetobacter skin carriage among US army soldiers deployed in Iraq. Infect Control Hosp Epidemiol. 2007;28: 720–722. 17520547 10.1086/518966

[86] Griffith ME, Ellis MW, Murray CK. Acinetobacter nares colonization of healthy US soldiers. Infect Control Hosp Epidemiol. 2006;27: 787–788. 16807863 10.1086/505923

[87] Simor AE, Lee M, Vearncombe M, Jones-Paul L, Barry C, Gomez M et al An outbreak due to multiresistant *Acinetobacter baumannii* in a burn unit: risk factors for acquisition and management. Infect Control Hosp Epidemiol. 2002;23: 261–267. 12026151 10.1086/502046

[88] Morgan DJ, Liang SY, Smith CL, Johnson JK, Harris AD, Furuno JP et al Frequent multidrug-resistant *Acinetobacter baumannii* contamination of gloves, gowns, and hands of healthcare workers. Infect Control Hosp Epidemiol. 2010;31: 716–721. 10.1086/653201 20486855 PMC3010849

[89] Jawad A, Seifert H, Snelling AM, Heritage J, Hawkey PM. Survival of *Acinetobacter baumannii* on dry surfaces: comparison of outbreak and sporadic isolates. J Clin Microbiol. 1998;36: 1938–1941. 9650940 10.1128/jcm.36.7.1938-1941.1998 PMC104956

[90] Dettenkofer M, Seegers S, Antes G, Motschall E, Schumacher M, Daschner FD. Does the architecture of hospital facilities influence nosocomial infection rates? A systematic review. Infect Control Hosp Epidemiol. 2004;25: 21–25. 14756214 10.1086/502286

[91] Zanetti G, Blanc DS, Federli I, Raffoul W, Petignat C, Maravic P et al Importation of *Acinetobacter baumannii* into a burn unit: a recurrent outbreak of infection associated with widespread environmental contamination. Infect Control Hosp Epidemiol. 2007;28: 723–725. 17520548 10.1086/517956

[92] Bogaerts P, Naas T, El Garch F, Cuzon G, Deplano A, Delaire T et al GES extended-spectrum β-lactamases in *Acinetobacter baumannii* isolates in Belgium. Antimicrob Agents Chemother. 2010;54: 4872–4878. 10.1128/AAC.00871-10 20805394 PMC2976171

[93] Wybo I, Blommaert L, De Beer T, Soetens O, De Regt J, Lacor P et al Outbreak of multidrug-resistant *Acinetobacter baumannii* in a Belgian university hospital after transfer of patients from Greece. J Hosp Infect. 2007;67: 374–380. 18023922 10.1016/j.jhin.2007.09.012

